# SIGIRR, a Negative Regulator of TLR/IL-1R Signalling Promotes Microbiota Dependent Resistance to Colonization by Enteric Bacterial Pathogens

**DOI:** 10.1371/journal.ppat.1003539

**Published:** 2013-08-08

**Authors:** Ho Pan Sham, Emily Yi Shan Yu, Muhammet F. Gulen, Ganive Bhinder, Martin Stahl, Justin M. Chan, Lara Brewster, Vijay Morampudi, Deanna L. Gibson, Michael R. Hughes, Kelly M. McNagny, Xiaoxia Li, Bruce A. Vallance

**Affiliations:** 1 Division of Gastroenterology, BC's Children's Hospital, the Child and Family Research Institute and the University of British Columbia, Vancouver, British Columbia, Canada; 2 Department of Immunology, Lerner Research Institute, Cleveland Clinic Foundation, Cleveland, Ohio, United States of America; 3 Department of Biology, University of British Columbia Okanagan, Kelowna, British Columbia, Canada; 4 Department of Medical Genetics, Biomedical Research Center, University of British Columbia, Vancouver, British Columbia, Canada; University of Toronto, Canada

## Abstract

Enteric bacterial pathogens such as enterohemorrhagic *E. coli* (EHEC) and *Salmonella* Typhimurium target the intestinal epithelial cells (IEC) lining the mammalian gastrointestinal tract. Despite expressing innate Toll-like receptors (TLRs), IEC are innately hypo-responsive to most bacterial products. This is thought to prevent maladaptive inflammatory responses against commensal bacteria, but it also limits antimicrobial responses by IEC to invading bacterial pathogens, potentially increasing host susceptibility to infection. One reason for the innate hypo-responsiveness of IEC is their expression of Single Ig IL-1 Related Receptor (SIGIRR), a negative regulator of interleukin (IL)-1 and TLR signaling. To address whether SIGIRR expression and the innate hypo-responsiveness of IEC impacts on enteric host defense, *Sigirr* deficient (−/−) mice were infected with the EHEC related pathogen *Citrobacter rodentium*. *Sigirr −/−* mice responded with accelerated IEC proliferation and strong pro-inflammatory and antimicrobial responses but surprisingly, *Sigirr −/−* mice proved dramatically more susceptible to infection than wildtype mice. Through haematopoietic transplantation studies, it was determined that SIGIRR expression by non-haematopoietic cells (putative IEC) regulated these responses. Moreover, the exaggerated responses were found to be primarily dependent on IL-1R signaling. Whilst exploring the basis for their susceptibility, *Sigirr −/−* mice were found to be unusually susceptible to intestinal *Salmonella* Typhimurium colonization, developing enterocolitis without the typical requirement for antibiotic based removal of competing commensal microbes. Strikingly, the exaggerated antimicrobial responses seen in *Sigirr −/−* mice were found to cause a rapid and dramatic loss of commensal microbes from the infected intestine. This depletion appears to reduce the ability of the microbiota to compete for space and nutrients (colonization resistance) with the invading pathogens, leaving the intestine highly susceptible to pathogen colonization. Thus, SIGIRR expression by IEC reflects a strategy that sacrifices maximal innate responsiveness by IEC in order to promote commensal microbe based colonization resistance against bacterial pathogens.

## Author Summary

Despite being in close contact with billions of commensal bacteria, the epithelial cells that line the intestine develop very weak innate inflammatory responses to bacterial products. The goal of this study was to explore why these cells respond so poorly, and how increasing their innate responsiveness would impact on host defense against invading bacterial pathogens. We show that a negative regulator of innate signaling called SIGIRR, limits the inflammatory responses of the intestine to bacteria. Following infection by the bacterial pathogen *Citrobacter rodentium*, the intestines of mice lacking SIGIRR showed exaggerated inflammatory, antimicrobial and proliferative responses. Through transplantation studies, we showed it was SIGIRR expression by intestinal epithelial cells that limits these responses, and that the exaggerated responses were driven by cytokine signaling through the interleukin-1 receptor. Despite their exaggerated responses, SIGIRR deficient mice proved extremely susceptible to infection by *C. rodentium* and other intestinal bacterial pathogens. We found the exaggerated inflammatory responses rapidly depleted intestinal commensal microbes, reducing their ability to outcompete invading pathogens for space and nutrients (colonization resistance). Our study thus clarifies that the hypo-responsiveness of epithelial cells plays an unexpected but critical role in host defense, by promoting commensal microbe based competition against enteric pathogens.

## Introduction

*Citrobacter rodentium* is a mouse-specific attaching/effacing (A/E) bacterial pathogen related to the clinically important enteropathogenic *Escherichia coli* (EPEC) and enterohemorrhagic *E. coli* (EHEC). *C. rodentium* has been widely used to define the *in vivo* virulence strategies employed by A/E pathogens [1], [2]. It has also proven a popular model to assess host immune responses against mucosal bacterial pathogens as well as explore how these pathogens compete with intestinal commensal microbes for colonization niches and nutrients [3], [4]. We have shown that Myeloid differentiation factor (MyD) 88 dependent innate receptors play a critical role in driving the host response to infection as well as controlling *C. rodentium* burdens carried by mice [5]. Moreover, we and others have shown that infected *Myd88* deficient (−/−) mice suffer severe intestinal epithelial cell (IEC) damage leading to widespread ulcers and necrosis of their colonic tissues. MyD88 signaling thus appears to limit tissue damage during infection by this pathogen, potentially by promoting increased IEC proliferation [5], [6].

While the full array of receptors involved in the protective actions of MyD88 in this model is still unclear, TLR4 appears to drive the inflammatory response as well as promote IEC proliferation. We previously showed that infected *Tlr4 −/−* mice displayed only modest increases in colonic crypt heights and were attenuated in the induction of the chemokines MCP-1 and MIP2α, as well as the recruitment of neutrophils and macrophages to the infected colon [7]. Despite these defects in their inflammatory responses, *Tlr4 −/−* mice were still able to clear *C. rodentium* infection. In contrast, TLR2 and interleukin (IL)-1R both protect IEC integrity and drive IEC repair [2], [7], [8], [9]. Infected *Tlr2 −/−* mice suffer severe ulceration in their mid and distal colons. Surprisingly, their exaggerated pathology was not due to higher *C. rodentium* burdens, as colonization levels were similar to wildtype mice [10], but instead involved impaired IEC barrier function and reduced production of the cytokine IL-6, leading to exaggerated IEC apoptosis during infection. Similarly, mice lacking the IL-1R were found to be more susceptible to *C. rodentium* infection, developing exaggerated mucosal damage following challenge [8].

Since many of the MyD88 dependent responses to *C. rodentium* infection involve changes in IEC function or proliferation, we asked whether they reflected MyD88 dependent signalling within the IEC themselves. While it is clear that MyD88 signalling can occur within IEC, in general IEC are hypo-responsive to most bacterial products and other aspects of inflammatory signalling [11]. Since IEC exist in close proximity to trillions of commensal bacteria, their hypo-responsiveness may be necessary to maintain a mutualistic relationship with the resident microbiota and prevent spontaneous intestinal inflammation [2]. The commensal microbiota, aside from providing nutrients to the host, are important contributors to host defense by promoting resistance to colonization by enteric pathogens [12]. Disruption of the commensal microbiota (through antibiotic treatment, for example) enhances enteric pathogen colonization and subsequent disease in mice [13], [14], [15]. However, the molecular mechanisms that underlie commensal microbe based resistance to colonization in the mammalian intestine remain poorly defined.

Recent studies suggest that the innate hypo-responsiveness of IEC may in part reflect the actions of Single Ig IL-1 related receptor (SIGIRR) (also called TIR8), a negative regulator of IL-1R and TLR signaling [16], [17]. SIGIRR suppresses MyD88 dependent signaling by sequestering IL-1 receptor-associated kinase (IRAK)1 and TNF receptor associated factor (TRAF) 6, both downstream targets of MyD88 [17], [18], [19]. Our laboratory recently demonstrated that stable overexpression of SIGIRR within IEC lines diminished NF-κB–mediated IL-8 responses to TLR ligands and to IL-1β. In contrast, suppression of SIGIRR by small interfering ribonucleic acid (siRNA) significantly enhanced Caco-2 and HT-29 cell line production of IL-8 in response to these ligands [20]. We have also shown that *Sigirr* −/− mice suffer extensive mucosal damage and increased mortality during dextran sodium sulfate (DSS) induced colitis [21]. The protective role of SIGIRR in this model was shown to reflect the suppression of inflammatory signalling within IEC, such that in the absence of SIGIRR, IEC exhibited exaggerated inflammatory responses driven by the presence of the commensal microbiota. Despite these findings, the specific receptors regulated by SIGIRR were not defined [21]. To address what impact increased innate responsiveness by IEC would have on intestinal host defense against pathogenic bacteria, we tested the effects of SIGIRR on the host response to *C. rodentium* infection.

As previously reported, the intestines of uninfected *Sigirr* −/− mice were histologically similar to those of WT mice [21], [22] but following *C. rodentium* infection, *Sigirr −/−* mice developed exaggerated inflammatory and antimicrobial responses, along with increased mucosal damage and IEC proliferation. Bone marrow (BM) transplantation experiments confirmed that loss of SIGIRR in the non-BM compartment (putative IEC) controlled the exaggerated colitic and IEC responses. These responses appeared largely independent of TLR2 or TLR4 but were instead dependent on IL-1R signalling. Despite their exaggerated colitic and antimicrobial responses, *Sigirr −/−* mice showed dramatically increased susceptibility to *C. rodentium* infection, in association with the rapid depletion of their intestinal commensal microbes. This loss of commensals reduced the host's resistance to colonization by *C. rodentium* as well as to other enteric bacterial pathogens. Thus SIGIRR plays an unexpected role in enteric host defense, promoting commensal microbe-based colonization resistance to pathogens at the expense of limiting antimicrobial, colitic and IEC homeostatic responses.

## Results

### MyD88 signaling in IEC plays little role in the host response to *C. rodentium* infection

**Figure 1:**
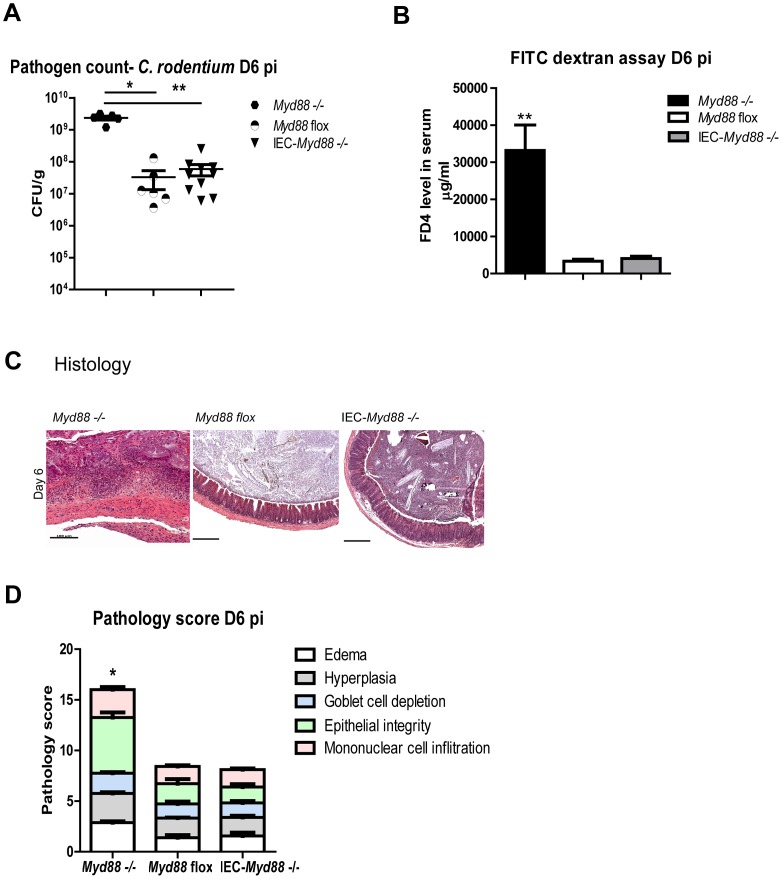
MyD88 signaling in IEC is not required for protection against *C. rodentium* infection. *Myd88* −/−, *Myd88* flox and IEC-*Myd88* −/− mice were infected for 6 days with *C. rodentium*. Infected IEC-*Myd88* −/− mice carried similar (A) pathogen burdens, (B) levels of serum FD4, and (C–D) similar mucosal damage as *Myd88* flox mice. Moreover all of these readouts are significantly greater in *Myd88* −/− mice, as compared to *Myd88* flox and IEC-*Myd88* −/− mice. Pathogen counts represent mucosal associated bacteria. Results are pooled from 2 independent infections with n = 3–4 mice per group. Error bars = SEM, (Student t test *P<0.05, ** P<0.01). Images were taken at 200× magnification.

MyD88 signaling plays a critical role in the development of protective host responses to *C. rodentium* infection, controlling pathogen burdens, driving inflammation and promoting IEC integrity/proliferation [5], [6]. As a result, *Myd88* −/− mice are highly susceptible to *C. rodentium* infection, carrying heavy pathogen burdens and suffering severe mucosal damage and loss of IEC barrier function (Figure 1A–1D). Since many of these MyD88 dependent protective responses involve changes in IEC function, we tested whether they reflected MyD88 signaling within the IEC. Crossing *Myd88* flox mice with mice expressing the cre enzyme under the villin (IEC specific) promoter, we generated IEC-*Myd88* −/− mice that were then infected with *C. rodentium* along with control *MyD88* flox mice. Interestingly, MyD88 signaling within IEC had little impact on the host response to infection. Pathogen burdens in the IEC-*Myd88* −/− mice were similar to those in control mice (Figure 1A), as were crypt heights and tissue integrity. Moreover, we noted no overt difference in IEC barrier function or inflammatory cell recruitment to the infected cecum between these two mouse strains (Figure 1B–1D). These data indicate that MyD88 signaling within IEC plays little role in driving the innate host response to *C. rodentium* infection.

### *Sigirr −/−* mice develop exaggerated colitis during *C. rodentium* infection

**Figure 2:**
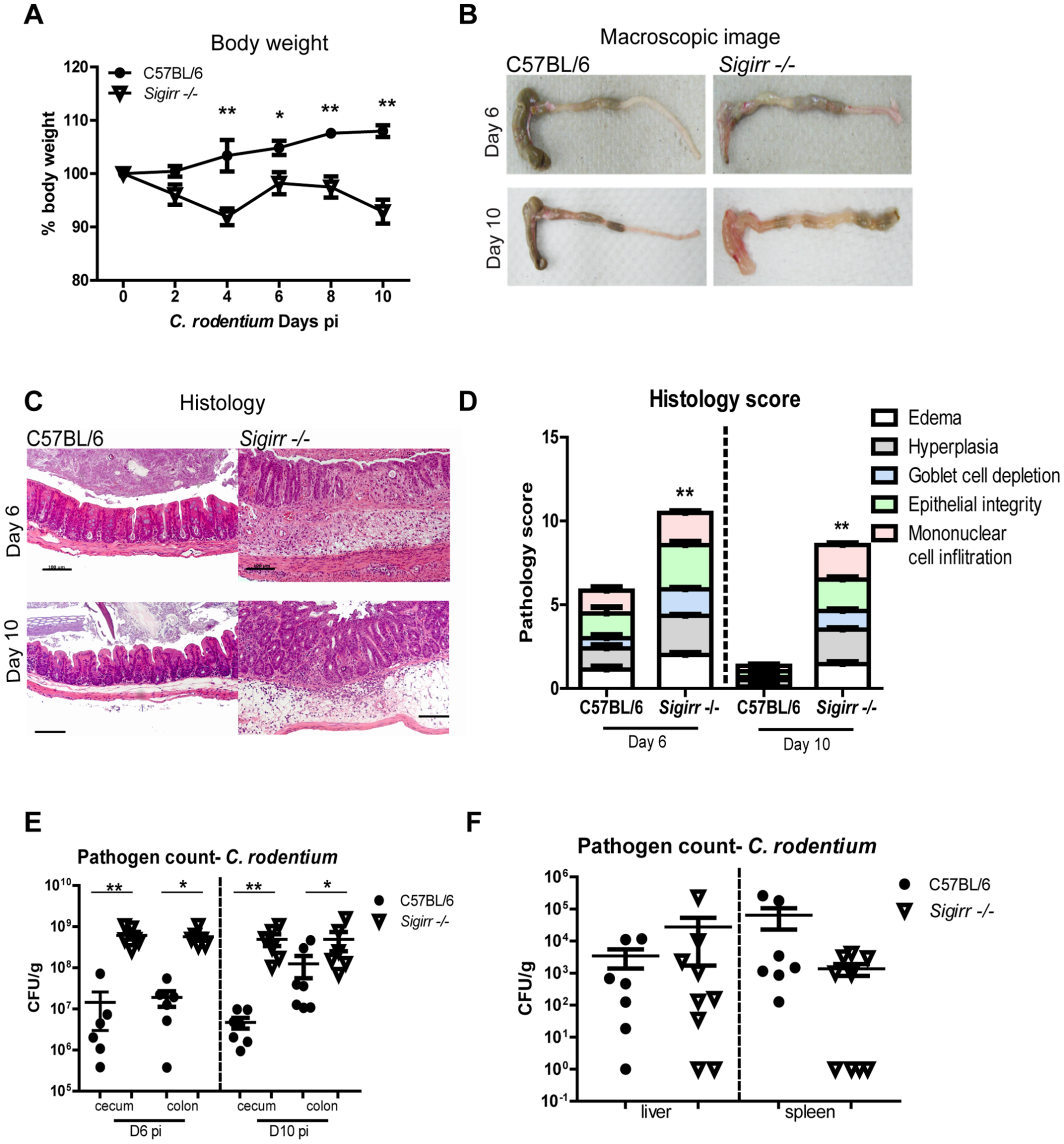
*Sigirr* −/− mice suffer more severe colitis during *C. rodentium* infection. *Sigirr −/−* mice exhibited (A) rapid weight loss by D4 pi. At both D6 and D10 pi, (B–C) their ceca displayed severe damage, with loss of stool contents and focal ulcers. (D) Cecal tissues from *Sigirr −/−* mice had significantly higher pathology scores at D6 and D10 pi compared to WT mice. Plating revealed *Sigirr −/−* mice carried significantly higher pathogen burdens than WT mice in (E) cecal and colonic tissues, but their burdens were similar in (F) liver or spleens. Pathogen counts represent mucosal associated bacteria. Results are pooled from 2 independent infections with n = 3–4 per group. Error bars = SEM, (Two-way ANOVA (Figure A), Student t test (Figure D, E, and F, *P<0.05, **P<0.01). Images were taken at 200× magnification.

While it is unclear why IEC play such a limited role in driving MyD88 dependent responses to *C. rodentium* infection and other forms of colitis [23], [24], [25], we recently showed that SIGIRR, a negative regulator of TLR and IL-1R signaling, is expressed by IEC and limits their responses to IL-1β and to most bacterial PAMPs [20]. To test the impact of SIGIRR *in vivo*, *Sigirr* −/− mice were infected with *C. rodentium* under co-housing conditions (WT and *Sigirr −/−* mice in same cage) or with WT and *Sigirr −/−* mice housed in different cages, and similar results were obtained. As shown in Figure 2A, infected WT mice displayed modest weight loss at day (D) 2 post-infection (pi) followed by recovery and progressive weight gain. In contrast, infected *Sigirr* −/− mice exhibited significantly greater (P<0.05) weight loss (∼10%) starting from D4 until D10 pi (Figure 2A). Following their euthanization, the *Sigirr* −/− mice showed greater macroscopic signs of infection than WT mice, with overt loss of stool content and edema seen throughout their large intestines (Figure 2B). Moreover, pus and blood were often found within the ceca of infected *Sigirr −/−* mice and between 60 and 80% of these mice suffered macroscopic cecal ulcers at D6 and D10 pi, a phenotype not observed in WT mice.

Based on the observed pathology, as well as the fact that baseline SIGIRR gene transcript levels in WT mice were highest in the cecum (not shown), we focused our subsequent analysis on the cecum. At both D6 and D10 pi, *Sigirr* −/− mice showed significantly higher cecal pathology scores compared to WT mice, reflecting increased crypt hyperplasia, edema, and greater inflammatory cell infiltration (Figure 2C and 2D, P<0.05). Specifically, we noted increased macrophage and neutrophil infiltration into the cecal mucosa of *Sigirr* −/− mice. Similarly, gene transcript levels for chemokines (MCP-1 and MIP-2α), antimicrobial factors (mCRAMP, β-defensin III, and Reg-3γ) and cytokines (IL-17A, TNF-α and IFN-γ) were all significantly elevated at D6 and D10 pi in *Sigirr* −/− mice compared to WT mice (Figure S1). Based on their exaggerated colitic responses, we expected *Sigirr* −/− mice would show some level of protection against infection. Instead, they carried significantly higher (10–100 fold) *C. rodentium* burdens in their ceca and colons compared to WT mice at both D6 and D10 pi (Figure 2E, P<0.01). Interestingly, these higher burdens reflected increased numbers of *C. rodentium* infecting the intestinal mucosal surface, as well as within the intestinal lumen. Notably, despite their high intestinal pathogen burdens, *Sigirr −/−* mice did not show increased susceptibility at systemic sites, as *C. rodentium* burdens in the spleens and livers of *Sigirr −/−* mice were similar to those in WT mice (Figure 2F).

### *Sigirr −/−* mice exhibit a hyper-proliferative IEC response against *C. rodentium* infection

**Figure 3:**
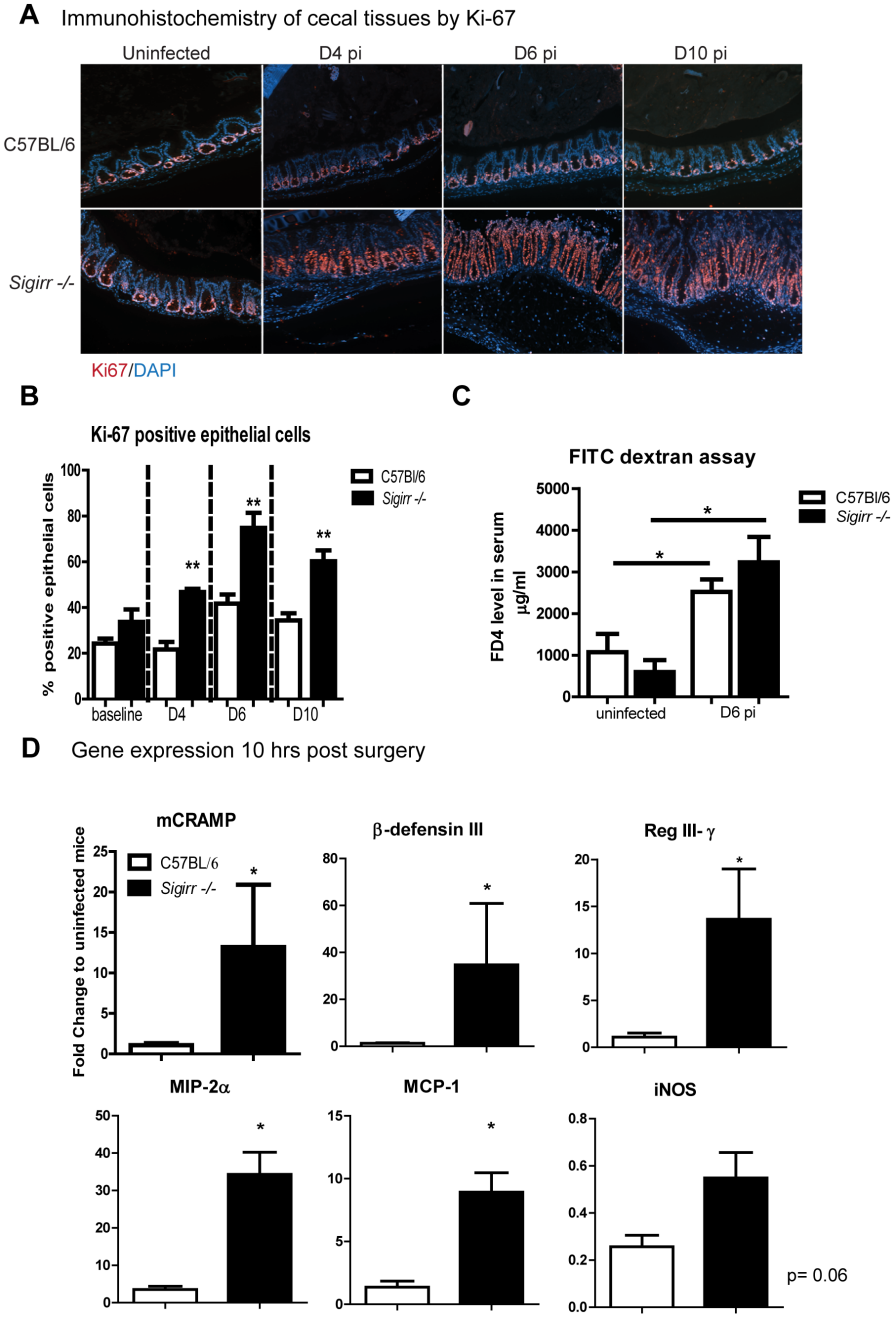
*Sigirr −/− mice* exhibit stronger inflammatory responses during *C. rodentium* infection. Immunostaining for the proliferation marker (A) Ki-67 (red) revealed *Sigirr −/−* mice exhibit increased IEC proliferation in cecal tissues by D4 pi. (B) At D4, D6 and D10 pi, there are significantly more proliferating IEC in *Sigirr −/−* mice compared to WT mice. (C) WT and *Sigirr −/−* mice experience similar levels of barrier permeability following infection. (D) *Sigirr −/−* mice carry significantly higher gene transcript levels for antimicrobial peptides and chemokines compared to WT mice following cecal loop surgery. Results are representative of 4 independent infections with n = 3–4 per group. Error bars = SEM, (Student t test (Figure B and C), Mann-Whitney t test (Figure D), *P<0.05, **P<0.01).

Despite carrying heavy *C. rodentium* burdens and suffering exaggerated colitic damage, *Sigirr* −/− mice survived infection and healed their mucosal ulcers by D14 pi (Figure S2), suggesting that intestinal mucosal repair was enhanced in these mice. We assessed IEC proliferation in *Sigirr* −/− and WT mice (by Ki-67 staining) to examine their ability to repair mucosal damage and also measured IEC barrier integrity. Increased IEC proliferation, (as measured by the percentage of IEC in a crypt showing Ki-67 positive staining) was evident by D4 pi in the *Sigirr* −/− mice, but not in WT mice (Figure 3A and 3B, 47±1% vs 21±3%, P<0.01). By D6 pi, IEC proliferation in *Sigirr* −/− mice was significantly greater than in WT mice (Figure 3A and 3B, 75±7% vs 41±4%, P<0.01) and similar results were observed at D10 pi (Figure 3A and 3B, 60±5% vs 34±4%, P<0.01). We also assessed IEC barrier function by oral fluorescein isothiocyanate dextran (FD4) gavage, and while infection did cause an increase in serum FD4 levels in both strains, we noted no differences in FD4 levels between infected *Sigirr* −/− mice and WT mice (Figure 3C).

### The negative regulator SIGIRR suppresses the host response to *C. rodentium* infection

We next examined whether SIGIRR impacted on very early host responses to *C. rodentium* by employing the cecal ligation model of acute *C. rodentium* infection, allowing us to inject equal pathogen numbers into the ceca of WT and *Sigirr −/−* mice. As expected, similar numbers of *C. rodentium* were found infecting the cecal tissues of WT and *Sigirr* −/− mice after 10 hr of infection (WT: 1.9±0.7×10^8^ CFU/g, *Sigirr −/−*: 1.2±0.5×10^8^ CFU/g). Cecal tissues were collected and qPCR was used to determine transcript levels for genes encoding several antimicrobial peptides and other IEC derived factors. As shown in Figure 3D, infection of WT mice led to a modest increase in the transcription of genes encoding antimicrobial peptides (mCRAMP, Reg3γ, β-defensin III) and chemokines (MCP1 and MIP2α). In contrast, we observed strikingly elevated transcription of most genes in infected *Sigirr −/−* mice (P<0.05), despite the two mouse strains carrying similar pathogen burdens. These results indicate that in the absence of SIGIRR, the host intestine responds very rapidly to *C. rodentium* infection, and that the exaggerated colitis suffered by orally infected *Sigirr −/−* mice likely reflects both their lack of SIGIRR, as well as their increased pathogen burdens.

### SIGIRR's actions reflect its expression by non-BM derived cells

**Figure 4:**
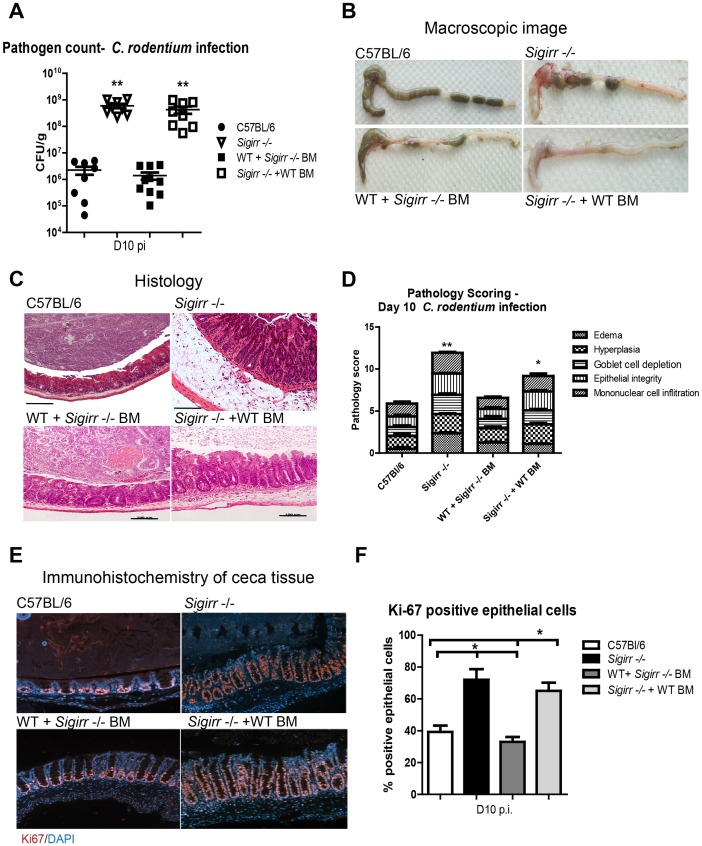
Non-BM derived cells mediate SIGIRR-dependent mucosal responses. WT and *Sigirr−/−* mice were used to generate BM chimeric mice, which were then infected for 10 days with *C. rodentium*. Similar to *Sigirr −/−* mice, *Sigirr −/−* + WT BM mice displayed significantly heavier (A) pathogen burdens compared to WT mice. The ceca of *Sigirr −/−* + WT BM mice displayed (B) severe macroscopic and (C) histologic damage with significantly (D) greater pathology scores compared to WT and WT + *Sigirr−/−* BM mice. *Sigirr −/−* + WT BM mice exhibit higher levels of IEC proliferation as revealed by (E and F) Ki-67 staining. Pathogen counts represent mucosal associated bacteria. Results are pooled from 2 independent infections with n = 3–4 per group. Error bars = SEM, (Student t test (Figure A and D), One way ANOVA with Bonferroni posttest for (Figure F), *P<0.05, **P<0.01). Images were taken at 200× magnification.

SIGIRR thus plays a critical role in suppressing *C. rodentium* induced colitis, but the cellular source of SIGIRR is unclear. SIGIRR is expressed by IEC, as well as by several BM-derived cell types including dendritic cells and T cells [17], [18]. To better define the cell type(s) regulated by SIGIRR during *C. rodentium* infection, we generated haematopoietic chimeras by BM transplantation between *Sigirr* −/− mice and WT mice expressing Ly5.1 on their BM-derived cells. Following infection by *C. rodentium*, *Sigirr* −/− + WT BM chimeric mice developed severe colitis, similar to that seen in *Sigirr* −/− mice. Moreover the ceca of infected WT + *Sigirr* −/− BM mice were macroscopically similar to those of WT mice, although histologically, they displayed a greater increase in crypt lengths (Figure 4B–4D) than WT mice. Pathology scoring also revealed *Sigirr* −/− + WT BM mice had exaggerated crypt hyperplasia, edema and immune cell infiltration when compared to WT + *Sigirr* −/− BM mice (Figure 4D, P<0.05). Similar to *Sigirr* −/− mice, *Sigirr* −/− + WT BM chimeras carried significantly higher intestinal *C. rodentium* burdens, compared to WT mice and WT + *Sigirr* −/− BM mice at D10 pi (Figure 4A). Furthermore, *Sigirr* −/− + WT BM mice exhibited hyper-proliferative IEC responses (65±5%) similar to those of *Sigirr* −/− mice (72±6%), as revealed by Ki-67 staining while the IEC proliferative response was similar between WT mice (39±4%) and WT + *Sigirr* −/− BM mice (33±3%) (Figure 4E and 4F). Thus expression of SIGIRR by non-BM derived cells plays the major role in suppressing the host inflammatory response and controlling *C. rodentium* burdens. Moreover, loss of SIGIRR in this compartment leads to the exaggerated colitis suffered by *C. rodentium* infected *Sigirr* −/− mice.

### Survival of infected *Sigirr −/−* mice requires MyD88, but not TLR2 or TLR4 signaling

**Figure 5:**
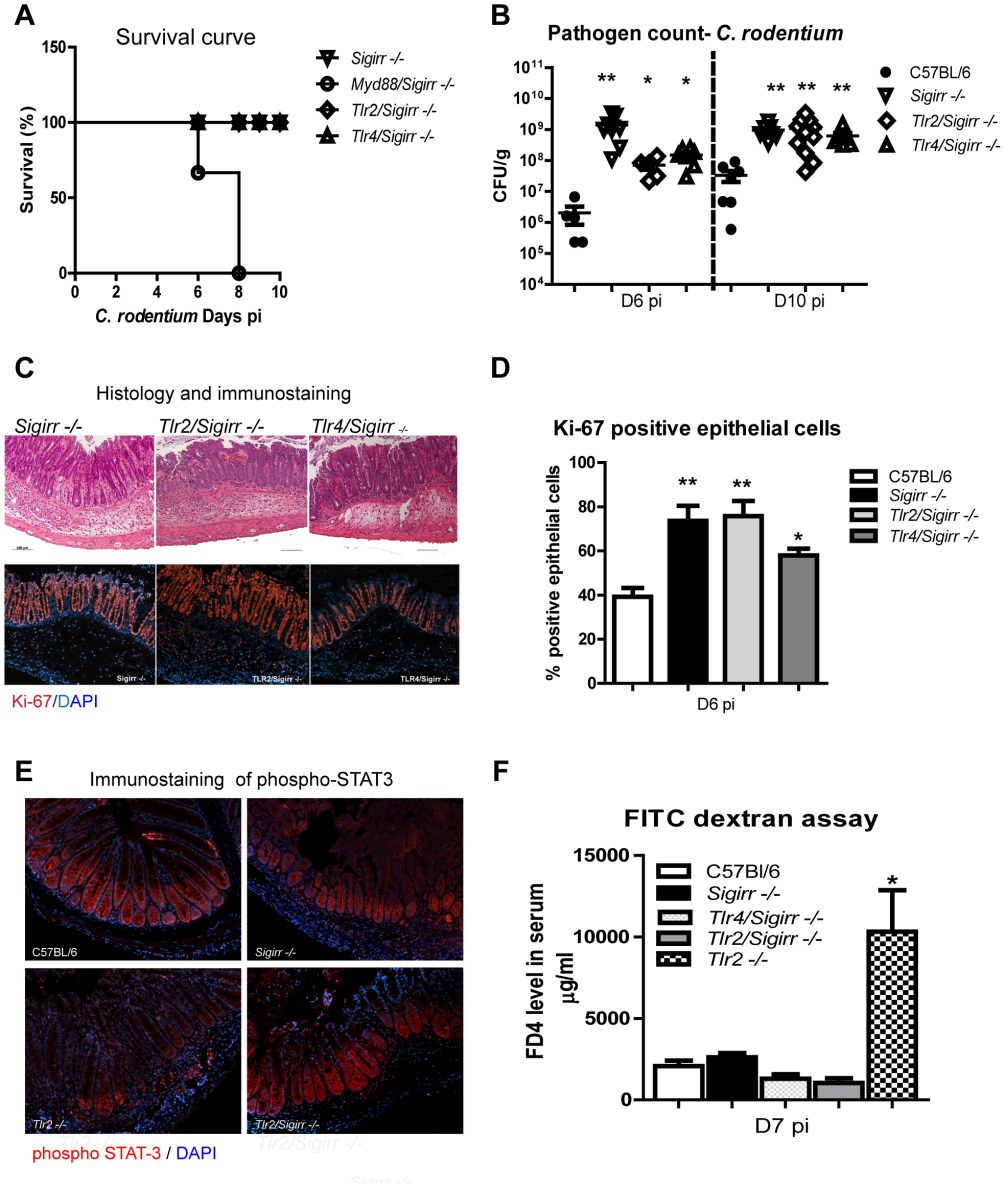
MyD88 signaling is required for the survival of infected *Sigrr −/−* mice. WT, *Sigirr −/−*, *Myd88/Sigirr −/−*, *Tlr2/Sigirr −/−*, and *Tlr4/Sigirr −/−* mice were infected by *C. rodentium* for 6 and 10 days. (A) *Myd88/Sigirr −/−* mice required euthanization by D8 pi whereas the other mouse groups survived the infection. (B) All mice on a *Sigirr −/−* background carried significantly heavier pathogen burdens and (C) showed more severe colitis compared to WT mice at D6 and D10 pi. (C–D) Immunostaining for the proliferation marker Ki-67 demonstrated infected *Sigirr−/−*, *Tlr2/Sigirr −/−* and *Tlr4/Sigirr −/−* display elevated IEC proliferation compared to WT mice. (E) phospho STAT-3 staining is restored in *Tlr2/Sigirr −/−* mice, while (F) the heightened barrier disruption seen in infected *Tlr2−/−* mice is normalized in *Tlr2/Sigirr −/−* mice. Pathogen counts represent mucosal associated bacteria. Results are pooled from 2–3 independent infections, each with n = 3–4 per group. Error bars = SEM, (Student t test (Figure B, D) and one way ANOVA (Figure D), *P<0.05, **P<0.01). Images were taken at 200× magnification.

SIGIRR is a negative regulator of TLR and IL-1R signaling, but the identity of the (unregulated) receptor(s) that drive the exaggerated colitis and IEC proliferative responses in infected *Sigirr −/−* mice is unclear. To identify the receptor(s) involved, we began by removing MyD88 dependent signaling from *Sigirr −/−* mice. We crossbred *MyD88* −/− mice and *Sigirr* −/− mice and infected the resulting *MyD88/Sigirr* −/− mice. Similar to *MyD88* −/− mice, *MyD88/Sigirr* −/− mice became sick and required euthanization by D6-8 pi (Figure 5A). They also carried heavy *C. rodentium* burdens (∼10^10^ CFU/g) at D6 pi and suffered severe mucosal damage and increased intestinal permeability much like *Myd88* −/− mice (not shown), confirming SIGIRR's actions in this model depends on MyD88 signaling.

Next, we sought to identify the specific TLRs responsible for the exaggerated colitis seen in *Sigirr* −/− mice. Previous studies have shown that the host response to *C. rodentium* infection is largely dependent on TLR2 and TLR4 [7], [10]. Therefore, we crossbred *Tlr2 −/−* mice and *Tlr4* −/− mice with *Sigirr* −/− mice to generate *Tlr2/Sigirr* −/− mice and *Tlr4/Sigirr* −/− mice. Upon infecting these mice along with WT and *Sigirr* −/− mice, and *Tlr2 −/−* and *Tlr4 −/−* mice, all mouse strains were found to survive the infection (Figure 5A and Figure S3). As expected, all readouts were dramatically greater in the *Sigirr* −/− mice, as compared to WT mice. Interestingly, *Tlr2 −/−* and *Tlr4 −/−* mice developed modestly elevated pathology compared to WT mice but much less than that suffered by *Sigirr −/−* mice (Figure S3). Moreover, the responses in *Tlr2/Sigirr* −/− mice and *Tlr4/Sigirr* −/− mice were similar to those in the *Sigirr* −/− mice including pathogen burdens and pathological damage (Figure 5B and 5C). Hence, the data suggest that the exaggerated damage and increased pathogen burden observed in the *Sigirr −/−* mice are largely independent of TLR2 and TLR4.

### SIGIRR controls IEC proliferation and integrity largely independent of TLR2 and TLR4

In previous studies, we showed TLR2 and TLR4 play specific and critical roles in controlling IEC responses during *C. rodentium* infection [9]. TLR4 signaling drives IEC proliferation during infection, while TLR2 signaling promotes IEC integrity. In fact, infected *Tlr2* −/− mice suffer exaggerated IEC barrier permeability, and impaired phospho-STAT3 signaling within their IEC, with these defects leading to cecal and colonic ulcers and high mortality rates [10]. When we assessed IEC responses in the *Tlr2/Sigirr* −/− mice and *Tlr4/Sigirr* −/− mice, the *Tlr2/Sigirr* −/− mice exhibited similar proliferative responses (74±7%, Figure 5D) to *Sigirr* −/− mice (75±7%) as demonstrated by Ki-67 staining. Interestingly, *Tlr4/Sigirr* −/− mice showed an intermediate level of IEC proliferation (58±3%), modestly but not significantly reduced compared to *Sigirr −/−* mice, yet still significantly greater (P<0.05) than WT mice (36±4%). This data suggests that signaling through other SIGIRR-regulated receptors drives IEC proliferation in *Sigirr −/−* mice, even in the absence of TLR4.

We next tested IEC barrier function in these mice (and *Tlr2* −/− mice). At D6 pi, FD4 serum levels within *Tlr2* −/− mice were significantly elevated compared to WT mice, confirming their impaired barrier function (Figures 5F and S3, P<0.05). Similar to WT mice and *Tlr4 −/−* mice, *Sigirr* −/− mice showed no overt barrier dysfunction, but strikingly, the concurrent loss of SIGIRR (*Tlr2/Sigirr* −/− mice) compensated for the barrier dysfunction seen in *Tlr2* −/− mice. Furthermore, the impaired phospho STAT-3 staining seen in *Tlr2* −/− mice was restored in *Tlr2/Sigirr −/−* mice (Figure 5E). These results indicate that loss of SIGIRR leads to IEC responses that promote mucosal integrity in a manner largely able to compensate for the defects caused by TLR2 or TLR4 deficiency, suggesting another SIGIRR regulated receptor is at play.

### Exaggerated IEC responses in *Sigirr −/−* mice require IL-1R signaling

**Figure 6:**
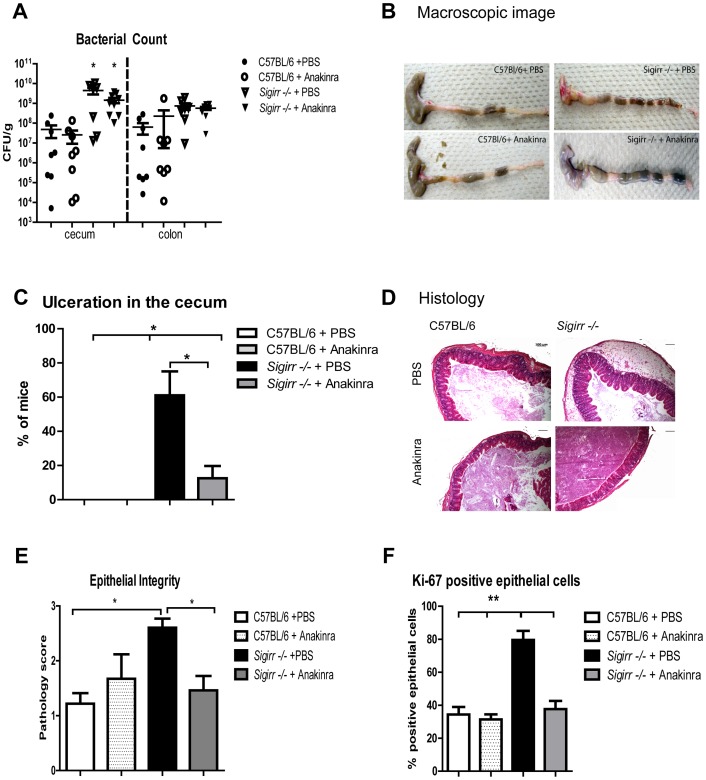
The exaggerated colitis seen in the *Sigirr −/−* mice is attenuated by treatment with the IL-1R antagonist anakinra. WT and *Sigirr −/−* mice were intra-peritoneally injected twice with anakinra (1 mg/mouse) each day for 6 days, or with PBS during *C. rodentium* infection. Anakinra treatment did not impact on pathogen burdens in infected mice (A) however treatment with anakinra ameliorated macroscopic ulceration in infected *Sigirr −/−* mice (B–C) along with reducing edema and improving epithelial integrity (D–E). Immunostaining for the proliferation marker Ki-67 demonstrated treatment with anakinra reduced the elevated IEC proliferation observed in *Sigirr −/−* mice (F). Pathogen counts represent mucosal associated bacteria. Results are pooled from 2 independent infections each with n = 3–4 per group. Error bars = SEM, (Student t test (Figure A, C), *P<0.05, **P<0.01). Images were taken at 200× magnification.

Aside from negatively regulating TLRs, SIGIRR also negatively regulates IL-1R and IL-18R signaling [17], [19], [26]. We tested *Il-18/Sigirr −/−* mice, and found no significant differences with *Sigirr −/−* mice in their colitic responses (not shown). To test whether IL-1R signaling contributes to the exaggerated colitic responses observed in infected *Sigirr −/−* mice, we first treated WT and *Sigirr −/−* mice with anakinra, an IL-1R antagonist [27]. While treatment with anakinra did not impact pathogen burdens (Figure 6A), it lessened the macroscopic damage that developed in *Sigirr −/−* mice compared to vehicle treated *Sigirr −/−* mice (Figure 6B and C). Histological pathology scoring revealed treatment with anakinra improved the epithelial integrity of the *Sigirr −/−* mice (Figure 6D–E and Figure S4). It also abrogated the exaggerated IEC proliferative response observed in *Sigirr −/−* mice (Figure 6F).

**Figure 7:**
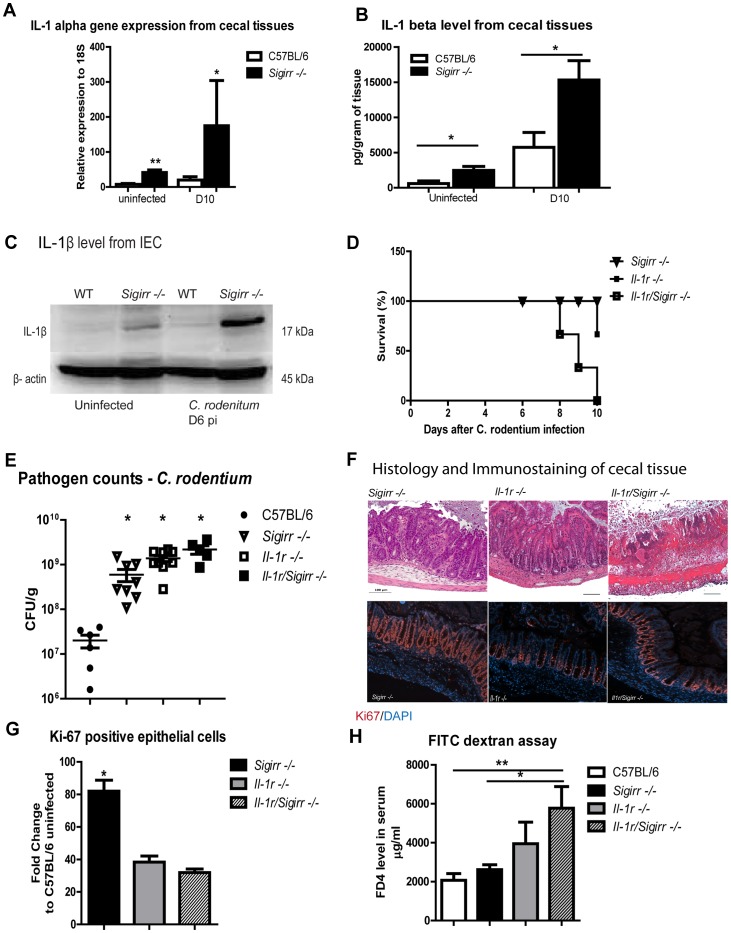
IL-1R signaling is required for the exaggerated IEC responses in *Sigirr −/−* mice. The cecal tissues from infected *Sigirr −/−* mice show increased abundance of IL-1α gene transcripts (A) as compared to WT mice. The *Sigirr −/−* mice also express increased levels of IL-1β protein in their cecal tissues under uninfected and *C. rodentium* infected conditions as measured by (B) ELISA and (C) Western blot. *Il-1r/Sigirr −/−* mice exhibit increased (D) mortality rates, (E) elevated pathogen burdens and (F) severe mucosal damage. The severe damage suffered by the *Il-1r/Sigirr −/−* mice was accompanied by impaired IEC proliferation as shown by (G) immunostaining for Ki-67 and by higher IEC permeability as quantified by (H) FD4 in serum. Pathogen counts represent mucosal associated bacteria. Results are pooled from 2–4 independent infections with n = 3–4 per group. Error bars = SEM, (Student t test (Figure A, B, E), one-way ANOVA (Figure G, H), *P<0.05, **P<0.01). Images were taken at 200× magnification.

To further explore the potential for IL-1R signaling to contribute to the exaggerated responses seen in infected *Sigirr −/−* mice, we assessed the expression of its ligands IL-1α and IL-1β. Interestingly, IL-1α gene expression showed a dramatic increase in the cecal tissues of *Sigirr −/−* mice during infection. Moreover protein analysis (ELISA) revealed significantly increased IL-1β levels in *Sigirr* −/− mice compared to WT mice under both baseline and infected conditions (Figure 7A and 7B, P<0.05). Similarly, cecal crypts isolated from *Sigirr* −/− mice showed elevated IL-1β levels under baseline and infected conditions as measured by Western blot analysis (Figure 7C).

Continuing to address the potential role of IL-1R signaling in the response of the *Sigirr* −/− mice, we generated *Il-1r/Sigirr* −/− mice and infected them, along with *Sigirr −/−* and *Il-1r* −/− mice with *C. rodentium*. The *Il-1r/Sigirr* −/− mice rapidly lost weight during infection, requiring their euthanization by D10 pi (Figure 7D). At D6 pi, we noted that although the *Sigirr* −/− mice, *Il-1r* −/− mice and *Il-1r/Sigirr* −/− mice all carried similar intestinal pathogen burdens, the *Il-1r/Sigirr* −/− mice suffered extensive mucosal damage compared to the *Sigirr* −/− mice and the *Il-1r* −/− mice (Figure 7E–F and Figure S5). Remarkably, the protective, hyper-proliferative IEC responses observed in the *Sigirr* −/− mice were absent in the *Il-1r/Sigirr* −/− mice (Figure 7G), with almost 3 fold more IEC staining positively for Ki-67 in the *Sigirr* −/− mice compared to the *Il-1r/Sigirr* −/− mice (72±6% vs 26±5%, P<0.01). Moreover, infected *Il-1r/Sigirr* −/− mice suffered significantly greater intestinal permeability compared to *Sigirr* −/−mice as measured by serum FD4 levels (Figure 7H, P<0.01), whereas the *Il-1r* −/− mice showed serum FD4 levels that were intermediate between the two other mouse strains. These findings indicate that the exaggerated and protective IEC responses seen in *Sigirr* −/− mice are largely IL-1R dependent and IL-1R may be the key receptor regulated by SIGIRR in this model.

### SIGIRR deficiency increases susceptibility to *C. rodentium* colonization/infection

**Figure 8:**
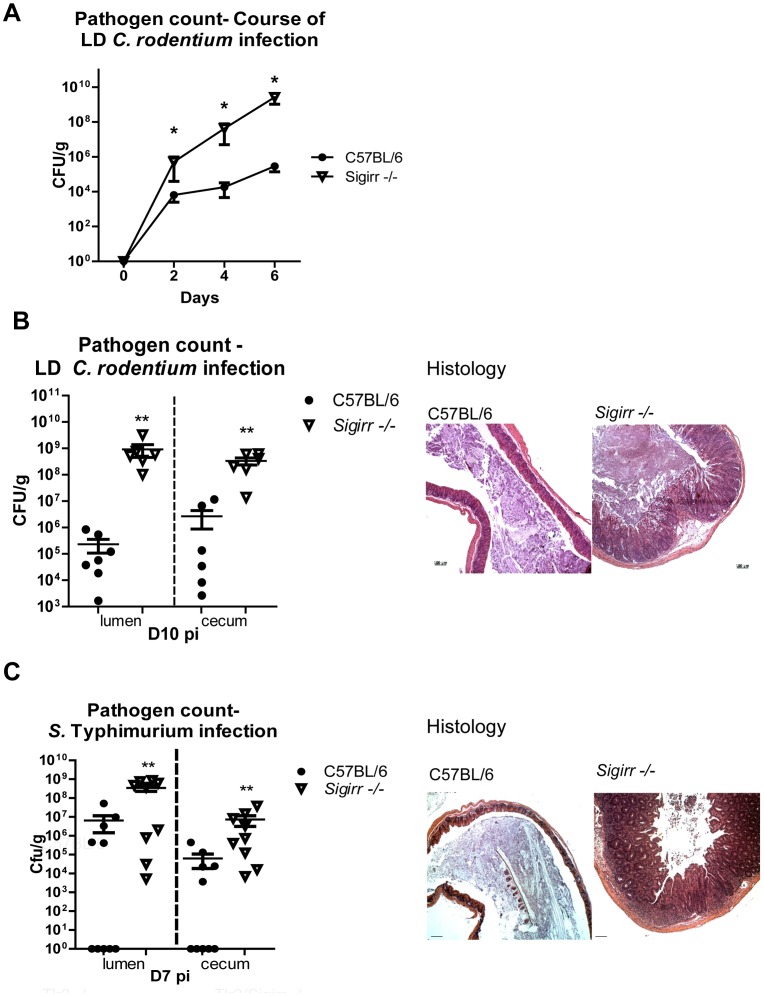
*Sigirr −/−* mice are highly susceptible to enteric infection. (A) *Sigirr −/−* mice were heavily colonized by 100× lower dose (LD) of *C. rodentium* by D2 pi. By D10 pi, *Sigirr −/−* mice carried 1000× heavier (B) pathogen burdens and developed severe mucosal damage compared to WT mice. (C) WT and *Sigirr −/−* mice infected by *S.* Typhimurium without streptomycin pre-treatment. By D7 pi, *Sigirr −/−* mice were heavily colonized and underwent extensive cecal injury. Pathogen counts represent mucosal associated bacteria. Results are pooled from 2–3 independent infections with n = 3 per group. Error bars = SEM, (Two-way ANOVA (Figure A), Student t test (Figures B and C), *P<0.05, **P<0.01)). Histological images were taken at 100× magnification.

While *Sigirr* −/− mice develop exaggerated colitic and IEC proliferative/reparative responses during infection, these mice also appear more susceptible to *C. rodentium* colonization, carrying significantly higher pathogen burdens than mice expressing SIGIRR. To test whether *Sigirr −/−* mice are truly more susceptible to *C. rodentium* infection, we infected WT and *Sigirr* −/− mice with a 100 fold lower dose (LD) of *C. rodentium* and followed their course of infection by plating feces. Previous studies have found this low dose is unable to effectively infect WT mice [28], and indeed, LD WT mice shed only low numbers of *C. rodentium* (10^4^–10^6^ CFU/g of feces). In contrast, LD *Sigirr* −/− mice showed significantly (100×) higher *C. rodentium* burdens by D2 pi and their pathogen burdens continued to increase until D6 pi when they reached ∼2.6×10^9^ CFU/g of feces, similar to the burdens carried by *Sigirr −/−* mice given a full infectious dose (Figure 8A). At D10 pi, following tissue collection, *Sigirr* −/− mice were found to carry 1000 fold more *C. rodentium* in both cecal and colonic tissues compared to WT mice (Figure 8B, P<0.01). As expected from their pathogen burdens, *Sigirr* −/− mice developed severe inflammation and cecal pathology similar to that seen with a full dose infection whereas WT mice did not display any overt or histological signs of infection or pathology (Figure 8B).

### *Sigirr −/−* mice are also highly susceptible to *S.* Typhimurium colonization/infection

Thus *Sigirr −/−* mice show unusual susceptibility to *C. rodentium* colonization and infection. We next tested whether they showed heightened susceptibility to infection by another enteric bacterial pathogen. We orally inoculated WT and *Sigirr* −/− mice with the enteric bacterial pathogen *Salmonella enterica serovar* Typhimurium. *S.* Typhimurium is known to poorly colonize the intestines of mice, in large part because it is unable to displace competing commensal microbes [15], [29], [30]. As a result, pretreating mice with the antibiotic streptomycin is commonly used to remove competing commensals and facilitate *S.* Typhimurium colonization and infection of the cecum [15]. While we found *Sigirr −/−* mice were more susceptible than WT mice to *S.* Typhimurium infection following streptomycin pretreatment (not shown), we also tested their susceptibility to *S.* Typhimurium in the absence of antibiotic pretreatment. As expected, oral inoculation of WT mice with a dose of 5×10^6^ CFU of *ΔaroA S.* Typhimurium did not lead to any significant cecal pathology or colonization by this pathogen. In contrast, the same dose led to dramatically heavier (1000 fold) *S.* Typhimurium burdens in the cecum and severe inflammation and pathology in the ceca of *Sigirr* −/− mice (Figure 8C), similar to that seen in *S.* Typhimurium infected mice pretreated with streptomycin. Taken together, these results show that loss of SIGIRR expression leads to dramatically enhanced susceptibility to oral infection by enteric bacterial pathogens, in a manner able to overcome the typical commensal microbe dependent resistance to such colonization.

### *Sigirr −/−* mice undergo rapid commensal microbe depletion following infection

**Figure 9:**
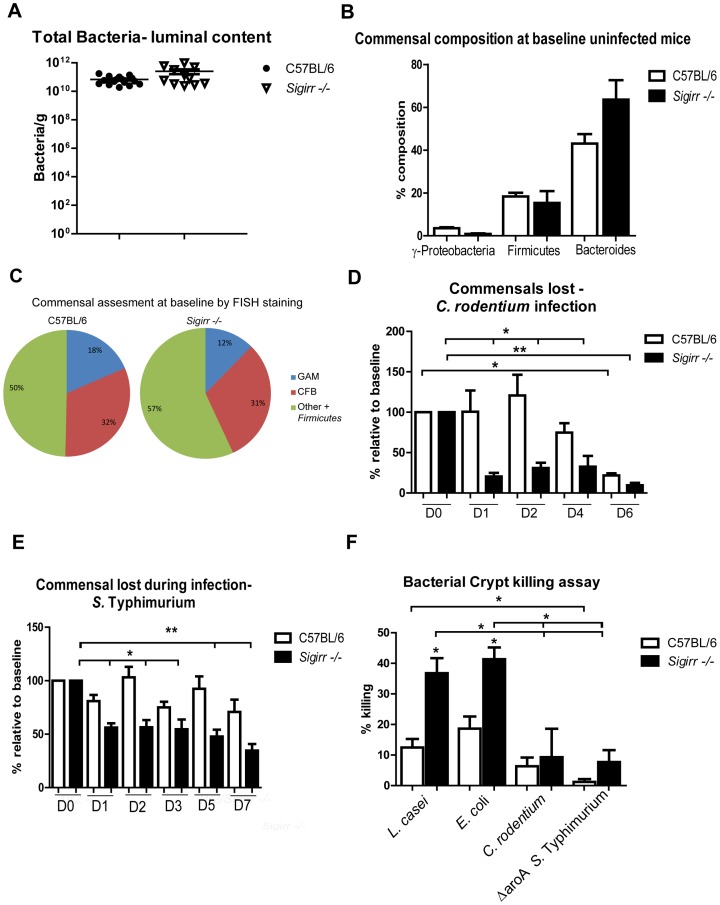
*Sigirr −/−* mice display strong antimicrobial activity against commensal microbes. No significant differences were found between (A) the commensal microbiota found in WT and *Sigirr −/−* mice as measured by (B) qPCR and (C) FISH staining. Rapid commensal depletion in *Sigirr −/−* mice was observed as early as D1 pi by *C. rodentium* (D) and *S.* Typhimurium (E) while intestinal crypts isolated from *Sigirr −/−* mice (F) possess greater killing activity against commensal *E. coli* and *Lactobacilli*. Results are pooled from 2–3 independent experiments (or infections) with n = 3–5 per group. Error bars = SEM, (Student t test (Figure A) one way ANOVA (Figures B–F, *P<0.05, **P<0.01).

Previous studies by our group and others found that differences in the makeup of the intestinal microbiota can affect host susceptibility to *C. rodentium* infection [31]. Previous sequencing studies found no overt differences between the microbiota of *Sigirr −/−* mice and WT mice [32], but considering the susceptibility of *Sigirr* −/− mice to enteric pathogens, we speculated they might instead carry either fewer total commensal microbes, or harbour a different microbial composition than WT mice. We enumerated the microbiota in the feces of these mice by fluorescence microscopy under baseline conditions, but noted no significant differences in commensal numbers between WT mice and *Sigirr* −/− mice (Figure 9A). Through qPCR analysis of fecal pellets, we found members of the phylum *Bacteroides* to be the dominant taxonomic group present within the stool of both *Sigirr −/−* and WT mice (∼50–60%), with smaller numbers of *Firmicutes* (∼20%), and other microbes making up minor components of the microbiota. We also looked at representative members of these phyla such as *Bifidobacteria*, *Lactobacillus* and *Clostridium* [33] and did not note any large or overt differences between the WT and *Sigirr −/−* mice (data not shown).

We also analyzed the above mentioned stool samples using fluorescence in situ hybridization (FISH). The FISH analysis relied on staining microbes with probes for Eubacteria as well as probes specific for *γ-Proteobacteria* or probes that recognize only three genera (*Cytophaga*, *Flexibacter* and *Bacteroides*) from the phylum *Bacteroides*. Microbes that stained with the Eubacteria probe, but did not stain with the other probes were placed in the “Other” category, with these microbes primarily encompassing the *Firmicutes* as well as minor components of the microbiota. While the makeup of the microbiota as determined by the FISH staining may be less accurate when compared to the more thorough qPCR approach (Figure 9B and 9C), both techniques were consistent in showing no statistically significant differences between uninfected *Sigirr −/−* and WT mice, confirming previous reports by Chan et al. [32]

Interestingly, Lupp et al. (2007), showed that *C. rodentium* infection causes a dramatic shift in the makeup of the gut microbiota as well as a reduction in total commensal numbers. These commensal changes were host mediated and took approximately 6 days to occur, corresponding with the time taken for *C. rodentium* to heavily colonize its host. We, and others have speculated these changes in the microbiota may facilitate *C. rodentium* colonization, as antibiotic based removal of commensals accelerates the course of *C. rodentium* infection [31], [33]. We quantified commensal populations within the feces of infected WT mice, and confirmed it takes approximately 6 days for the aforementioned commensal depletion (80%) to occur. In contrast, total commensal numbers in the fecal samples collected from the *Sigirr −/−* mice dropped dramatically by D1 pi, and this reduction remained evident until D6 pi and beyond (Figure 9D). The depletion of commensal microbes affected all relevant phyla to a roughly similar degree as measured by qPCR (data not shown). The corresponding *C. rodentium* burdens increased in concert (albeit slightly delayed) with the loss of commensals. Rapid depletion of total commensal microbe numbers was also detected in *Sigirr* −/− mice following infection with LD *C. rodentium* (not shown). Similarly, we also observed rapid depletion of total commensal microbe numbers by D2 post-*S.* Typhimurium infection (in the absence of antibiotics) (Figure 9E, *P<0.05). In contrast, this reduction in commensals was not observed in *S.* Typhimurium infected WT mice. Interestingly, we did not observe commensal depletion when *Sigirr −/−* mice were infected with an avirulent strain (*ΔescN*) of *C. rodentium* (not shown). These results suggest the impaired resistance to pathogen colonization we observed in *Sigirr −/−* mice reflects the ability of *C. rodentium* and *S.* Typhimurium to rapidly trigger commensal depletion in these mice, thereby opening niches within the intestine for rapid pathogen colonization.

### *Sigirr −/−* crypt epithelial cells show heightened antimicrobial activity

We next addressed the basis for the rapid depletion of commensal microbes seen in infected *Sigirr* −/− mice. Notably, the massive commensal depletion occurs prior to overt intestinal inflammation. As outlined in Figure 3D, acute exposure to *C. rodentium* rapidly increased antimicrobial gene expression, likely reflecting responses by IEC. We thus examined whether the IEC of *Sigirr* −/− mice harbored greater bactericidal activity than WT mice towards commensal bacteria, using commensal *E. coli* and *Lactobacilli caesi* as examples. We isolated intestinal crypts under baseline condition from WT and *Sigirr* −/− mice; collected their crypt supernatants and tested their bactericidal activity against the aforementioned commensal microbes. After a 1 hr incubation, isolates from *Sigirr* −/− mice showed a killing activity of 29.9±3.5% against commensal *E. coli* while crypt isolates from WT mice showed only 10.6±9.9%. Similarly, isolates from *Sigirr −/−* mice displayed greater killing activity than WT mice against *Lactobacilli caesi* (38.4±2.4% versus 23.4±5.4%) a gram-positive commensal microbe (P<0.05). Therefore, crypt isolates from *Sigirr* −/− mice possess significantly higher bactericidal activity than those from WT mice.

We next we sought to determine whether the bactericidal activity within the crypt isolates of *Sigirr −/−* mice increased during infection, and whether the enteric pathogens *C. rodentium* and *S.* Typhimurium were more resistant to this bactericidal activity compared to commensal microbes. We isolated intestinal crypts from WT and *Sigirr* −/− mice after 24 hrs of *C. rodentium* infection; collected their crypt supernatants and tested their bactericidal activity against both commensal microbes and pathogenic microbes. As shown in Figure 9F, while the killing of *L. casei* by *Sigirr −/−* crypt isolates was relatively unchanged from baseline, the killing of the commensal *E. coli* strain significantly increased to 41.4±3.9% at day 1 pi (P<0.05). Moreover, both *C. rodentium* and *S.* Typhimurium were found to be significantly more resistant to the bactericidal activity of the crypt isolates from both WT and *Sigirr −/−* mice compared to the commensal microbes (P<0.05). Considering the rapid induction of antimicrobial genes seen in the cecal loop model, the heightened antimicrobial activity of *Sigirr −/−* mice likely explains the rapid commensal depletion seen following their infection.

## Discussion

MyD88 dependent signaling plays a critical protective role during *C. rodentium* infection, promoting inflammatory and antimicrobial responses that control *C. rodentium* burdens, as well as homeostatic responses that protect IEC barrier function and limit/repair IEC injury [5], [6]. While many of these responses involve changes in IEC function, we clearly show that MyD88 signaling within IEC plays little role in driving these responses. Rather than IEC being unable to respond to *C. rodentium* infection, we instead show that the negative regulator SIGIRR suppresses their responsiveness. While *Sigirr −/−* mice react strongly to pathogenic insults to the GI tract, *Sigirr −/−* mice do not develop spontaneous colitis or other baseline pathologies, but instead show a modestly elevated inflammatory tone within their intestines [21]. When we infected *Sigirr −/−* mice, they developed exaggerated inflammatory and antimicrobial responses as well as increased IEC proliferation and repair. These responses began within the IEC layer, and through BM transplantation studies, we confirmed that it was SIGIRR expression by non-BM cells (putative IEC) that limits the host inflammatory and IEC responses to *C. rodentium*. This data thus expands our previous *in vitro* studies that the innate responsiveness of IEC to A/E bacterial pathogens is dramatically elevated in the absence of SIGIRR.

We previously showed *Sigirr −/−* mice develop exaggerated DSS colitis and IEC proliferation that depended on the presence of commensal microbes [21], but it was unclear which SIGIRR regulated receptors drove these responses. In the current study, we tested the role of MyD88, as well as individual receptors and as expected, loss of MyD88 left *Sigirr −/−* mice suffering severe mucosal damage and rapidly succumbing to infection, much like *Myd88 −/−* mice that express SIGIRR. As previously mentioned, TLR4 signaling drives the majority of inflammation and IEC proliferation during *C. rodentium* infection of SIGIRR expressing mice [9]. Similarly TLR2 signaling in these mice is required to protect and repair IEC barrier function during infection [9], [10]. Interestingly, neither TLR2 nor TLR4 were required for these roles in the absence of SIGIRR. In fact, SIGIRR deficiency led to increased IEC proliferation/barrier protection that was largely able to compensate for the defects caused by either TLR2 or TLR4 deficiency, suggesting another receptor was responsible for the protective IEC responses seen in *Sigirr −/−* mice.

Our data indicate that IL-1R signaling drives the exaggerated IEC responses seen in *Sigirr −/−* mice. While IL-1R signaling plays a protective role during *C. rodentium* infection of SIGIRR expressing hosts [8], it appears to be primarily responsible for the heightened IEC proliferation and barrier protection seen in *Sigirr −/−* mice, as these exaggerated IEC responses were attenuated following anakinra treatment, and lost in *Il-1r/Sigirr* −/− mice. While the increased pathogen burdens and heightened inflammation seen in the *Sigirr −/−* mice were not overtly IL-1R dependent, IL-1R signaling did prove crucial in limiting the severity of the resulting mucosal damage and ultimately for the survival of infected *Sigirr −/−* mice. Interestingly, we found elevated levels of both IL-1α and IL-1β in the intestinal tissues of *Sigirr* −/− mice under both baseline and infected conditions. Both IL-1α and IL-1β have been shown to induce IEC proliferation *in vitro*, as well as induce expression of chemokines and antimicrobial factors by IEC [34], [35], [36]. At present it is unclear which cytokine (or perhaps both) drives the exaggerated IL-1R dependent epithelial proliferative and repair responses seen in the *Sigirr −/−* mice.

Our data also indicates it is primarily SIGIRR expression by non-BM derived cells that limits the host response to *C. rodentium* infection. While several BM derived cell types (macrophages, dendritic cells, T cells) found in the intestine express SIGIRR, the only described non-BM derived cell type is the IEC [16], [17]. It has been speculated that maintaining the innate hypo-responsiveness of IEC helps prevent spontaneous commensal microbe driven intestinal inflammation, as seen in patients with Inflammatory Bowel Disease (IBD) [37]. While this may be the case in some situations, our data suggests that SIGIRR instead controls the threshold at which IEC respond to invading bacterial pathogens.

Notably, despite their innate hyper-responsiveness, *Sigirr −/−* mice proved highly susceptible to infection, undergoing very rapid pathogen colonization and ultimately carrying significantly heavier *C. rodentium* burdens than WT mice. Unlike *Myd88 −/−* mice where their immunodeficiency not only led to increased intestinal pathogen burdens, but also lethally high *C. rodentium* burdens in the liver and spleen, the *Sigirr −/−* mice only showed increased pathogen burdens in their ceca and colons. Heavy *C. rodentium* colonization of *Sigirr −/−* mice was evident between D2 and 4 pi, compared to the 6 days typically required for WT mice. Interestingly, we previously found that such rapid *C. rodentium* colonization was only seen in mice pretreated with the antibiotic streptomycin to displace commensal microbes. Taken together with the susceptibility of *Sigirr −/−* mice to *S.* Typhimurium infection without the typical requirement for streptomycin pretreatment, we focused on potential defects in commensals as underlying the susceptibility of *Sigirr −/−* mice to pathogen colonization of their intestines.

As outlined by Chan et al. [32], previous deep sequencing studies identified few differences between *Sigirr −/−* mice and WT mice in their baseline intestinal microbiota. In accordance with their results, we also found they carried similar numbers of intestinal commensal microbes, and showed only modest differences in their baseline microbiota makeup, as compared to WT mice. We therefore compared how the intestinal microbiota of *Sigirr −/−* and WT mice responded to *C. rodentium* infection. As shown by Lupp et al. [4], infection of WT mice leads to a host mediated depletion of the intestinal microbiota by D6 pi, concomitant with *C. rodentium* expansion and spread through the intestines of WT mice. We confirmed this timing in WT mice, whereas overt commensal depletion was found to occur by D1 pi in *Sigirr −/−* mice, and persisted thereafter. Though qPCR analysis, we found that the observed commensal depletion affected all commensal microbial phyla to a roughly similar degree. Interestingly, this rapid commensal depletion did not occur when *Sigirr −/−* mice were gavaged with avirulent *C. rodentium*, but it did occur when the mice were challenged with *S.* Typhimurium suggesting the commensal depletion results from a host response to active infection.

While infected *Sigirr −/−* mice do not display overt colitis at the time of commensal depletion, we showed that acute exposure to *C. rodentium* dramatically elevated mRNA transcript levels for a number of antimicrobial genes within the ceca of *Sigirr −/−* mice. This increased antimicrobial tone had a functional effect, as intestinal crypt IEC isolates from even uninfected *Sigirr −/−* mice displayed significantly greater killing activity against commensal *E. coli* and *Lactobacilli* bacteria than IEC from WT mice. We also demonstrated that the bactericidal activity of the *Sigirr −/−* crypt IEC selectively but significantly increased by as early as D1 pi, and that compared to the commensals, the pathogens *C. rodentium* and *S.* Typhimurium were comparatively resistant to killing by crypt IEC. Thus the rapid depletion of intestinal commensal microbes seen in infected *Sigirr −/−* mice likely reflects the heightened antimicrobial activity of their IEC and provides much of the basis for their reduced colonization resistance against enteric pathogens.

Colonization resistance was initially described by van der Waaij et al., [14] as the process whereby the intestinal microbiota protects itself as well as the host against incursion by new and often harmful microbes. While there is significant evidence for colonization resistance, such as the hyper-sensitivity of germfree mice to enteric infections, the exact mechanisms by which colonization resistance exerts its protective role are unclear [13], [14], [38]. Moreover, while such commensal mediated resistance to enteric pathogens is undoubtedly beneficial to the host, it is unclear whether host factors play a role in promoting colonization resistance. Our data suggests that it is in fact the innate hypo-responsiveness of IEC that is critical in promoting colonization resistance. While it may seem counter-intuitive that limiting host antimicrobial and inflammatory responses in the GI tract would be protective, as our LD infection data shows, in most cases when the host is exposed to small numbers of enteric bacterial pathogens, the resident microbiota are able to prevent the pathogens from successfully infecting the host. It appears that SIGIRR helps maintain host-commensal mutualism (and colonization resistance) in the face of noxious threats, such as the incursion by a bacterial pathogen, potentially by controlling the threshold at which the IEC respond.

Our results are intriguing since the exaggerated inflammatory/antimicrobial responses in the intestines of *Sigirr −/−* mice appeared to deplete only commensals and not *C. rodentium*. While our crypt killing assays suggest the responses elicited in *Sigirr −/−* mice are poorly effective against *C. rodentium* and *S.* Typhimurium, we believe the effects are more complex. It is well known that enteric bacterial pathogens utilize a number of strategies to subvert host defenses, including suppressing the inflammatory signaling of infected IEC [2], as well as possessing greater inherent resistance to the effects of antimicrobial peptides [39]. Moreover, recent studies have shown that enteric bacterial pathogens can utilize nutrients and metabolites released within the inflamed intestine that are unusable to commensal species [3], [40]. Therefore the success of bacterial pathogens at replacing the microbiota in *Sigirr −/−* mice may in fact also demonstrate their evolutionary success at withstanding host antimicrobial responses, and/or their ability to obtain nutrients within the intestinal lumen of the *Sigirr −/−* mice, in comparison to commensal microbes.

In conclusion, our data demonstrate that *Sigirr −/−* mice develop exaggerated intestinal inflammatory, antimicrobial and IEC proliferative responses to *C. rodentium* infection. While IL-1R signaling is key to many of the changes in IEC function and proliferation seen in these mice, there are other aspects of their exaggerated colitis (inflammation, commensal depletion etc) that are at least partially IL-1R independent but are still MyD88 dependent (not shown). We speculate these responses may reflect the combined actions of several TLRs since we were unable to identify a specific TLR responsible for these phenotypes. Moreover, although SIGIRR suppresses host inflammatory, antimicrobial and IEC reparative responses, it actually plays a critical role in enteric host defense by promoting commensal based colonization resistance against enteric bacterial pathogens. Thus SIGIRR's role in limiting the innate responsiveness of IEC is evidence of a complex defense strategy within the GI tract. This strategy delays the development of protective host responses to infection, by relying on cell types other than IEC to drive protective host (MyD88) responses to infection. However SIGIRR's key role in promoting and protecting host mutualism with the gut microbiota has obvious advantages as well, preventing many enteric infections by supporting the ability of the microbiota to resist invading microbes. While promoting colonization resistance to enteric infections is of great benefit, ultimately we believe that modulating SIGIRR expression within the intestine could also offer therapeutic potential for GI diseases, such as IBD. Increasing SIGIRR expression could potentially reduce inflammation in IBD patients, whereas suppressing SIGIRR could potentially help promote mucosal repair.

## Materials and Methods

### Mouse strains and infection of mice

Mouse strains used in this study: C57B/6 (WT), *Tlr2 −/−, Tlr4 −/−, Myd88 −/−, Sigirr* −/−, *Il-1r/Sigirr−/−*, *Tlr2/Sigirr* −/−, *Tlr4/Sigirr* −/− and *Myd88/Sigirr* −/− mice (8–12 weeks) were bred in house, and kept under specific pathogen free conditions at the Child and Family Research Institute. *Il-1r1−/−* and *Myd88* flox (B6.129P2-*Myd88^tm1Defr^*/J) and Villin cre mice were purchased from Jackson Laboratory. All double gene deficient mice were generated by crossbreeding single gene deficient mice. IEC-*Myd88* −/− mice were generated by crossbreeding *Myd88* flox mice with Villin-Cre mice. Mice were orally gavaged with an overnight culture of ∼2.5×10^8^ CFU of streptomycin resistant *C. rodentium* DBS100 or 1×10^6^ CFU of *ΔaroA S.* Typhimurium SL1344 (*ΔaroA* chosen because it does not kill Nramp1 sensitive mice) [41] and euthanized at specified time points pi. Infections were performed under both co-housing conditions (WT and mutant mice in same cage) and single housing conditions (WT and mutant in different cages). For IL-1R antagonist experiments, anakinra (Kineret) at 1 mg/mouse (or PBS as a control) was injected intraperitoneally twice daily (12 hrs apart) during the course of the infection (6 days) beginning on the day of the infection (day 0) just prior to oral gavage [42]. Body weight data are presented as the mean percentage of starting weight.

### Ethics statement

All experiments were performed according to protocols approved by the University of British Columbia's Animal Care Committee and in direct accordance with The Canadian Council on Animal Care (CCAC) guidelines. The protocols were approved by the UBC Animal Care committee (Protocols: A11-0290 and A11-0253). Mice were monitored for mortality and morbidity throughout their infection and euthanized if they showed signs of extreme distress or >15% body weight loss. All surgeries were performed under anesthesia (isofluorane and xylazine), and all efforts were made to minimize suffering.

### Tissue collection, bacterial counts and pathology scoring

Tissue collection and bacterial counts were performed as described previously [43]. Briefly, mice were anesthetized with isofluorane and euthanized over the course of infection, dissected and their large bowel was divided into cecum and colon to be collected in 10% neutral buffered formalin (Fisher) for histological analyses, or processed for tissue pathology assays. For viable cell counts, cecum, colon tissues and stool pellets were collected separately, and homogenized in PBS pH 7.4, with dilutions plated onto LB agar or streptomycin agar plates. Pathology was scored using a previously adapted scoring system [44]. In brief, paraffin-embedded cecal tissue sections (5 µm) that had been stained with haematoxylin and eosin were examined by two blinded observers. Tissue sections were assessed for i) submucosal edema (0- no change 1- mild 2- moderate 3- profound), ii) hyperplasia (0- no change 1-1-50% 2- 51%–100% 3 - >100%), iii) goblet cell depletion (0 no change 1 mild depletion 2 severe depletion 3- absence of goblet cells), iv) epithelial damage/integrity (0 no change 1- few cells sloughing, epithelial surface rippled 2- epithelial surface is rippled, damaged 3- epithelial surface is severely disrupted/damage, large amount of cells sloughing). For ulceration, an additional score of 1 was added for each 25% (or fraction of) of the tissue in the cross section affected ie.( a small ulcer would score as 4+1, whereas a tissue where 60% of the tissue cross section is ulcerated would be 4+3) v) mononuclear cells infiltration (per 40× field) (0- no change 1- <20 2- 20 to 50 3- >50 cells. The maximum possible score was 18.

### RNA extraction and quantitative real-time PCR

Following euthanization of mice, tissues were immediately transferred to RNA-later (Qiagen). Total RNA was purified using QiagenRNEasy kits (Qiagen) stored according to manufacturer's instructions. cDNA was synthesized with Omniscript RT kit (Qiagen) and OligodT (Applied Biological Material Inc) followed by quantitative real-time PCR techniques. qPCR was carried out on MJ Mini-Opticon Real-Time PCR System (Bio-Rad) using IQ SYBR Green Supermix (Bio-Rad) and MIP-2, MIP3-α, β-actin, mCRAMP, Reg 3-γ, β-defensin III primers using sequences and conditions previously described [44]. *Il-1α* primers were generated by PrimerBank (http://pga.mgh.harvard.edu/primerbank/), *5′* GAAGACTACAGTTCTGCCATT3′, reverse 5′ GACGTTTCAGAGGTTCTCAGAG 3′ Quantification was performed using the Gene Ex Macro OM 3.0 software (Bio-Rad) where PCR efficiencies for each primer set were incorporated into the final calculations.

### Immunofluorescence staining

Immunofluorescence staining of control and infected tissues was performed using previously described procedures [43]. In brief, paraffin embedded tissues were cut (6 µm) and were deparaffinized by heating at 55–65°C for 20 mins, cleared with xylene, rehydrated through an ethanol gradient to water. Sections were blocked using blocking buffer (Goat serum in PBS containing 1% bovine serum albumin (BSA), 0.1% Triton-X100, and 0.05% Tween 20, and 0.05% sodium azide). The primary antibodies used were Ki-67 (Thermo) or phospho STAT-3 (Ab-Cam) while the secondary was AlexaFluor 568-conjugated goat anti-rabbit IgG. Tissues were mounted using ProLong Gold Antifade reagent (Invitrogen) containing DAPI for DNA staining. Sections were viewed on a Zeiss AxioImager microscope and images taken using an AxioCam HRm camera operating through AxioVision software.

### Quantification of Ki-67 positive cells

Tissue were stained with Ki-67 and mounted with ProLong Gold Antifade with DAPI. Pictures were then taken for each tissue section. The number of Ki-67 positive cells and DAPI positive cells were counted for each crypt and calculated as a ratio of Ki-67 positive cells/DAPI positive cells as % positive cells. At least 6 crypts were counted from each section. The results expressed were averaged from at least 6 tissue sections.

### Cecal loop surgery

Cecal loop infections were performed as recently described [43]. In brief, mice were anaesthetized (2.5% isofluorane and xylazine at 10 mg/kg) and following a midline abdominal incision, the cecum was exposed. The proximal colon close to the cecum was ligated twice, while the ileocecal valve prevented backflow into the ileum, thus isolating the cecum. *C. rodentium* cultures were diluted in PBS, and 300 µl of culture containing ∼1×10^7^ CFU were injected into the cecal loop which was returned to the abdominal cavity and the incision closed with discontinuous sutures. The mice were euthanized at 10 hr pi and tissues and stool contents were collected for CFU counts and mRNA analysis as described above.

### Bone marrow transplantation

Bone marrow was isolated from C57BL/6.Ly5.1 (WT), or *Sigirr −/−* mice. Four million cells were intravenously injected into lethally irradiated wild-type (WT), or *Sigirr −/−* recipient mice that were allowed to recover for 12 weeks. Reconstitution was confirmed by FACs analysis of peripheral blood leukocytes for Ly5.1 expression. Only mice exhibiting greater than 90% chimerism were infected. Similar methods were performed to obtain *Sigirr −/−* +WT BM mice.

### Assessment of total microbes and FISH hybridization

Enumerating total microbes and FISH staining were performed as described previously [44].

At least 2 fecal pellets were collected from each animal at time points indicated. After homogenization, samples were placed in 10% Neutral Buffered Formalin to a final concentration of 3%. Samples were further diluted 1∶10 in PBS, vortexed briefly, and stored at 4°C. 2–5 µl of the 1∶10 diluted sample stored in PBS was diluted in 1 ml PBS and filtered onto Anodisc 25 filters (Whatman International Ltd) with a pore size of 0.2 µM and 2.5 cm diameter. The samples were allowed to thoroughly dry, and then were mounted on glass slides with ProLong Gold Antifade reagent containing DAPI (Molecular Probes) and viewed as above. The mean number of DAPI positive microbes counted in 3 to 6 randomly chosen fields per disc (1000×) was determined by two scorers. The total number of commensal microbes was calculated based on the mean numbers of all the counted fields and the dilution factor. The total number of commensal microbes was presented as the percentage of uninfected controls. For FISH staining, filters were passed through an ethanol gradient following filtering and samples were incubated overnight at 37°C in the dark with Texas red-conjugated EUB338 general bacterial probe (5′-GCT GCC TCC CGT AGG AGT-3′) and an AlexaFluor 488 conjugated GAM42a probe (5′-GCC TTC CCA CAT CGT TT-3′) that recognizes bacteria that belong to the γ-Proteobacter class [4], [45]. Filters were then washed in the dark with hybridization solution (0.9 M NaCL, 0.1 M TRIS pH 7.2, 30% Formamide, 0.1% SDS) for 15 min with gentle shaking. This step was repeated once with wash buffer (0.9 M NaCl, 0.1 M TRIS pH 7.2), and sections were placed in dH_2_O, and then mounted using ProLong Gold Antifade reagent with DAPI (Molecular Probes) and imaged as described above.

### Microbial composition analysis

Microbial composition was performed by qPCR as described previously [33]. Briefly, at least two fecal pellets were collected and DNA was extracted using a Qiagen DNA stool extraction kit. Following DNA extraction, 50 ng DNA/reaction was used for qPCR. qPCR was completed using 16 s rRNA group specific primers [33] to determine the relative abundance of selected bacterial phyla. The primers used were specific to the phyla *Bacteroides* (5′-GAG AGG AAG GTC CCC CAC-3′, 5′-CGC TAC TTG GCT GGT TCA G-3′) [46], and *Firmicutes* (5′-GGA GYA TGT GGT TTA ATT CGA AGC A-3′, 5′-AGC TGA CGA CAA CCA TGC AC-3′) [46], and *γ-Proteobacteria* (5′-TCG TCA GCT CGT GTY GTG A-3′, 5′-CGT AAG GGC CAT GAT G-3′) [47]. The relative abundance of each taxonomic group was determined by calculating the average Ct value relative to the total bacterial 16 s rRNA, as determined using the universal *Eubacteria* primers (5′-ACT CCT ACG GGA GGC AGC AGT-3′, 5′-ATT ACC GCG GCT GCT GGC - 3′) [48] and normalized to each primer's experimentally determined efficiency. Statistical significance was determined using a one-way ANOVA with a threshold of p<0.05.

### Colonic crypt killing assay

The bactericidal capacity of cecal crypt secretions was performed as described previously [49], [50]. Cecal and Colonic crypts were isolated from naïve WT and *Sigirr −/−* mice and mice infected with *C. rodentium* for 1 day. Ceca and colons were extracted from mice following euthanization and fecal contents were removed. Tissues were washed for 30 min at room temperature on an orbital shaker with HEPES buffered HBSS containing Pen/Strep and gentamycin. Tissues were then cut into small sections and placed in a petri dish containing 10 mL of BD cell recovery solution. Tissues were incubated for 2 hours at 4°C and crypts were dislodged from the tissues. Following centrifugation, crypts were resuspended in iPIPES buffer (10 mM PIPES; pH 7.4 and 137 mM NaCl), and counted. For experiments, 1000 crypts were incubated in 45 µL of iPIPES buffer at 37°C for 30 minutes. Following this period, 10 µL of a crypt-free isolate was added to 10^4^ *E. coli* (commensal isolates, a gift from Dr. Ben Willing) or *Lactobacili casei* (a gift from Dr. Alan Stintzi) or *C. rodentium* or *ΔaroA S.* Typhimurium and incubated for 37°C for 60 minutes. The antimicrobial capacity of crypt-culture extracts was assessed by counting the overnight growth of WT and *Sigirr −/−* crypt-treated *E. coli* or *Lactobacili casei*, and expressed as a percentage of the growth observed in untreated cultures.

### FD4 intestinal permeability assay

The assay was performed as previously described [9]. Uninfected or infected mice at D6 pi were gavaged with 150 µl of 80 mg/ml 4 kDa FITC-dextran (Sigma; FD4) in PBS 4 hrs prior to sacrifice. Mice were anaesthetized and blood (∼500 µl) was collected by cardiac punctures, which was added immediately to a final concentration of 3% acid-citrate dextrose (20 mM citric acid, 100 nM sodium citrate, 5 mM dextrose). Plasma was collected and fluorescence was quantified using a VictorX3 (Perkin-Elmer Life Sciences) at excitation 485 nm, emission 530 nm for 1 sec.

### Measurement of IL-1β in tissues

Tissues were collected as described above. After homogenization and centrifugation, tissue supernatants were collected at specified time points and IL-1β levels were assessed using an ELISA kit (BD Bioscience) following manufacturer's instructions.

### Western blotting

Following isolation of intestinal crypts, cells were lysed in RIPA buffer on ice. IEC proteins (50 µg) were resolved by 12% SDS-PAGE and transferred to PVDF membranes. Blots were blocked for 1 h with 5% milk in TBST. Membranes were incubated with primary antibody in TBST overnight at 4°C and probed with the respective secondary antibody for 1 hr at room temperature. Rabbit polyclonal IL-1β (Genetex) and mouse polyclonal actin (SantaCruz) antibodies were used in this study. Membranes were then incubated with ECL Prime Western Blotting kit (GE health) and imaged using a Biorad ChemiDoc XRS (BioRad)

### Statistical analysis

All results are expressed as the mean value ± SEM. Unless specified otherwise, non-parametric Mann–Whiney t-tests, or Student t tests were performed (Prism version 4.00). A *P* value of 0.05 or less was considered significant.

### Gene accession numbers

The following are the GeneIDs (Database: Entrez Gene) for each gene analyzed in this manuscript, given as gene name (official symbol **GeneID** #): TNF-α (Tnf **GeneID**: 21926); IL-1 α (Il1a **GeneID**: 16175); IL-1β (Il1b **GeneID**: 16176); IFN-γ (Ifng **GeneID**: 15978); IL-17A (Il17a **GeneID**: 16171); MCP-1 (Ccl2 **GeneID**: 20296); iNOS (Nos2 **GeneID**: 18126); mCRAMP (Camp **GeneID**: 12796); Mip2 (Cxcl2 **GeneID**: 12796); Reg-3γ (Reg3g **GeneID**: 19695); SIGIRR (SIGIRR **GeneID**: 24058)

## Supporting Information

Figure S1 *Sigirr −/− mice* exhibit higher inflammatory responses than WT mice during *C. rodentium* infection. (A) Quantitative PCR of mRNA from cecal tissues taken from WT and *Sigirr −/−* mice at D6 and D10 pi revealed *Sigirr −/−* mice undergo significantly greater induction of gene transcription for pro-inflammatory chemokine, cytokine and antimicrobial genes. Results are pooled from 2–3 individual infections with n = 3–5 per group. Error bars = SEM, (Student t test, *P<0.05)
(TIF)

Figure S2 *Sigirr −/−* mice recover from their severe colitis and mucosal ulceration by D14 pi following *C. rodentium* infection. Tissues from *Sigirr −/−* mice recover from *C. rodentium* infection and display similar tissue morphology as WT mice. Histological images were taken at 200×.
(TIF)

Figure S3 *Tlr2−/−, Tlr4 −/−* mice were infected with *C. rodentium* for 6 days. *Tlr2 −/− and Tlr4 −/−* mice harbour higher pathogen burdens (mucosal associated bacteria) (A) and suffer more severe mucosal damage than WT mice, but less than *Sigirr −/−* mice (B). *Tlr2 −/−* and *Tlr4 −/−* mice exhibited lower levels of epithelial proliferation compared to *Sigirr −/−* mice (B). *Tlr2 −/−* mice displayed increased barrier permeability as revealed by the FD4 assay (C). Results are pooled from 2–3 independent infections, with n = 3–4 per group. Error bars = SEM, (Student t test (Figure A, C), *P<0.05, **P<0.01). Images were taken at 200× magnification.
(TIF)

Figure S4 Total pathology score for anakinra treated mice infected with *C. rodentium* for 6 days. *Sigirr −/−* mice treated with anakinra underwent less pathological damage compared to *Sigirr −/−* mice treated with PBS control. Results are pooled from 2 independent infections each with n = 3–4 per group. Error bars = SEM (Student t test, *P<0.05,)
(TIF)

Figure S5 Total pathology score for *Il-1r* −/− and *Il-1r/Sigirr* −/− mice infected with *C. rodentium* for 6 days. *Il-1r/Sigirr −/−* mice exhibit increased pathological damage compared to *Sigirr −/−* and *Il1r −/−* mice. All three groups of mice suffered from greater damage compared to C57Bl/6 mice. Results are pooled from 2–4 independent infections with n = 3–4 per group. Error bars  =  SEM (Student t test, *P<0.05,)
(TIF)
