# Supplementary figures and images for: SIGIRR, a Negative Regulator of TLR/IL-1R Signalling Promotes Microbiota Dependent Resistance to Colonization by Enteric Bacterial Pathogens
Source: PLoS Pathog. 2013 Aug 8;9(8):e1003539. doi: 10.1371/journal.ppat.1003539 (PMC3738496; doi:10.1371/journal.ppat.1003539)

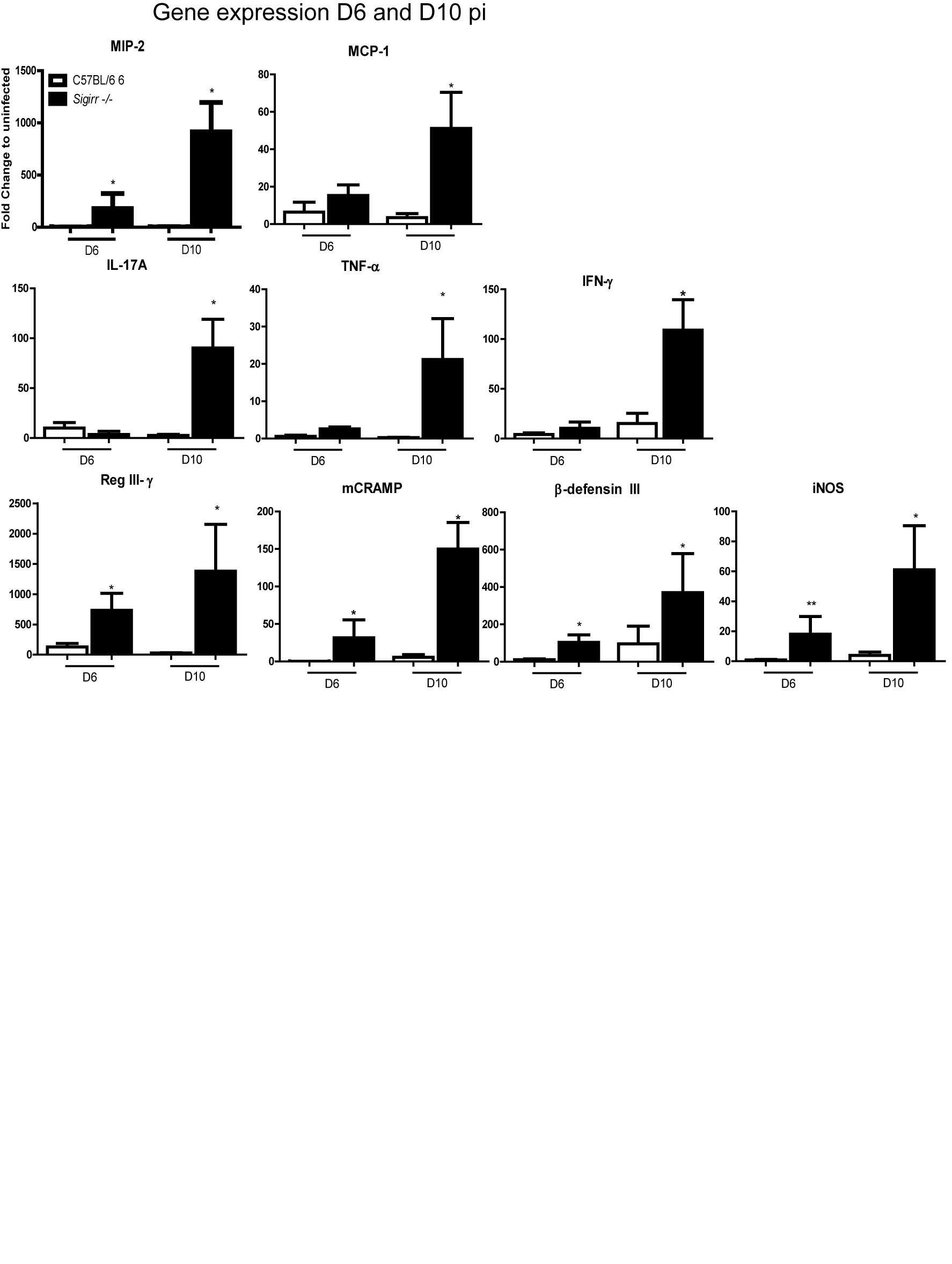

Supplement: Figure S1 — Sigirr −/− mice exhibit higher inflammatory responses than WT mice during C. rodentium infection. (A) Quantitative PCR of mRNA from cecal tissues taken from WT and Sigirr −/− mice at D6 and D10 pi revealed Sigirr −/− mice undergo significantly greater induction of gene transcription for pro-inflammatory chemokine, cytokine and antimicrobial genes. Results are pooled from 2–3 individual infections with n = 3–5 per group. Error bars = SEM, (Student t test, *P<0.05) (TIF) [file ppat.1003539.s001.tif]

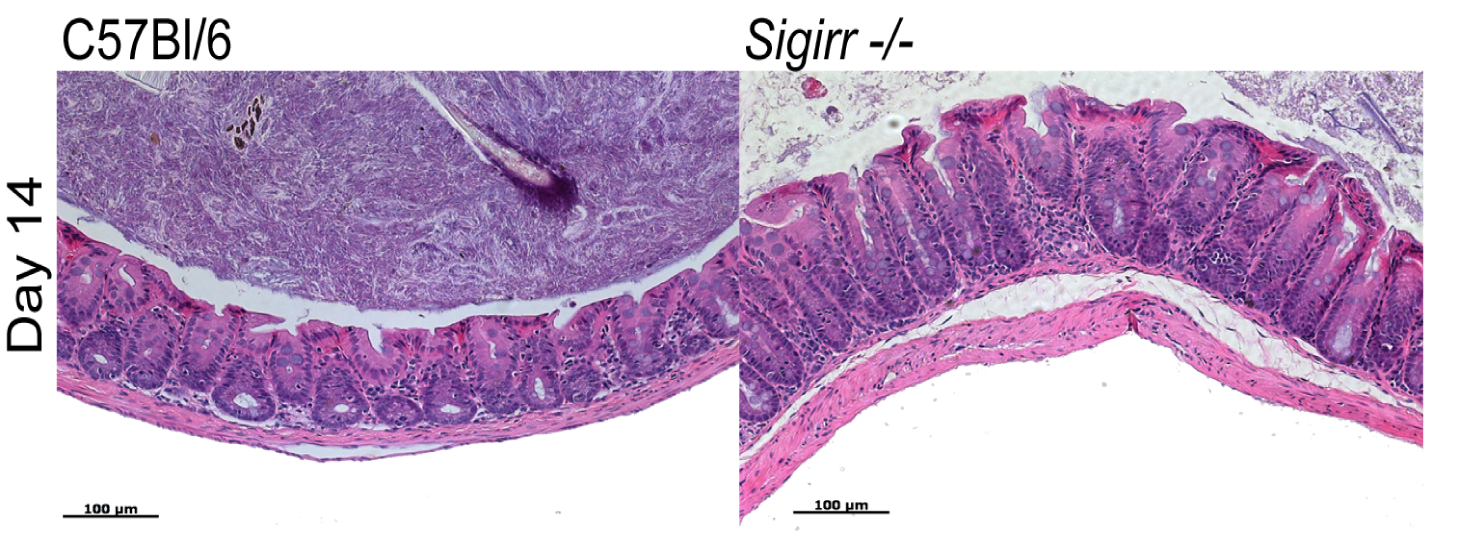

Supplement: Figure S2 — Sigirr −/− mice recover from their severe colitis and mucosal ulceration by D14 pi following C. rodentium infection. Tissues from Sigirr −/− mice recover from C. rodentium infection and display similar tissue morphology as WT mice. Histological images were taken at 200×. (TIF) [file ppat.1003539.s002.tif]

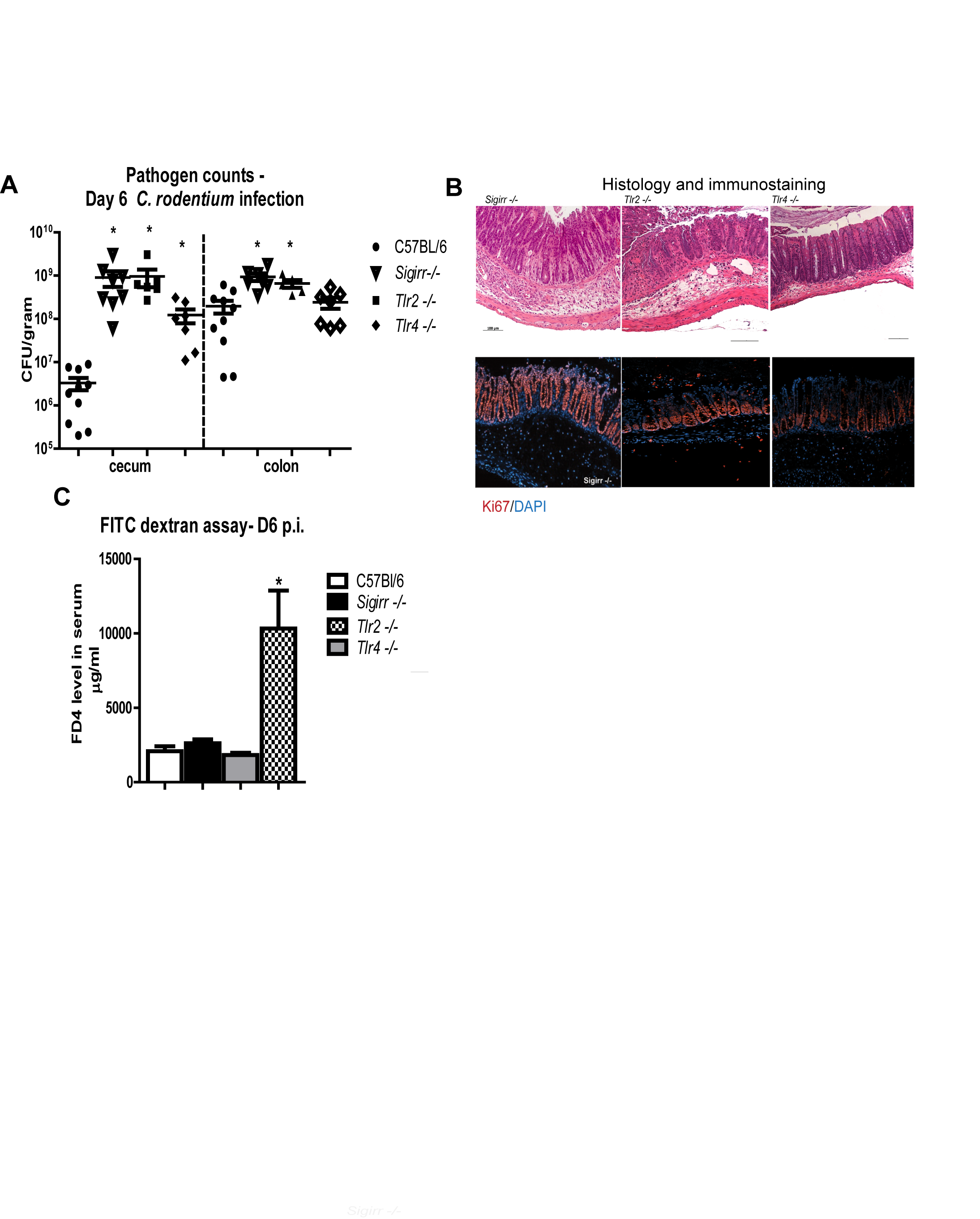

Supplement: Figure S3 — Tlr2−/−, Tlr4 −/− mice were infected with C. rodentium for 6 days. Tlr2 −/− and Tlr4 −/− mice harbour higher pathogen burdens (mucosal associated bacteria) (A) and suffer more severe mucosal damage than WT mice, but less than Sigirr −/− mice (B). Tlr2 −/− and Tlr4 −/− mice exhibited lower levels of epithelial proliferation compared to Sigirr −/− mice (B). Tlr2 −/− mice displayed increased barrier permeability as revealed by the FD4 assay (C). Results are pooled from 2–3 independent infections, with n = 3–4 per group. Error bars = SEM, (Student t test (Figure A, C), *P<0.05, **P<0.01). Images were taken at 200× magnification. (TIF) [file ppat.1003539.s003.tif]

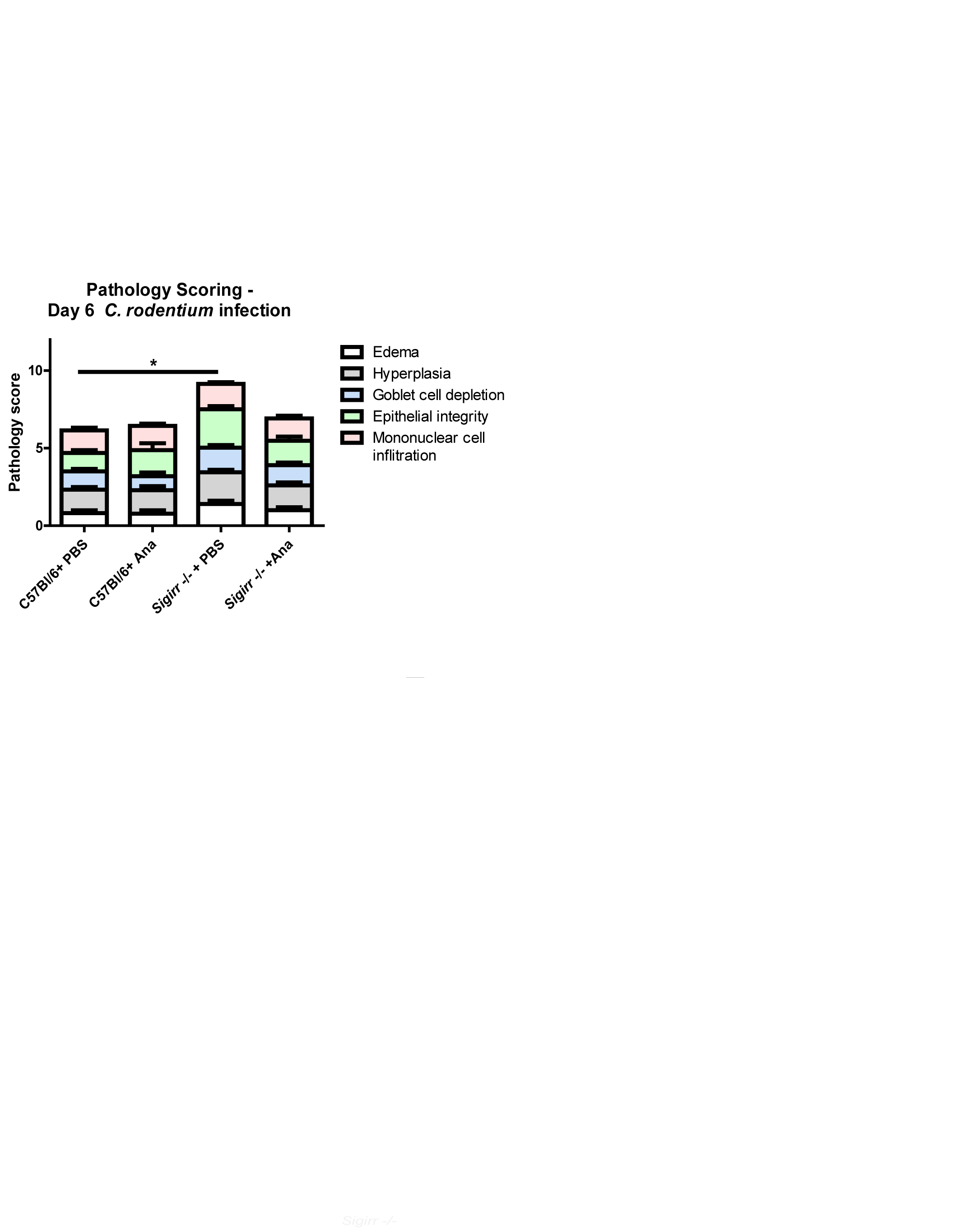

Supplement: Figure S4 — Total pathology score for anakinra treated mice infected with C. rodentium for 6 days. Sigirr −/− mice treated with anakinra underwent less pathological damage compared to Sigirr −/− mice treated with PBS control. Results are pooled from 2 independent infections each with n = 3–4 per group. Error bars = SEM (Student t test, *P<0.05,) (TIF) [file ppat.1003539.s004.tif]

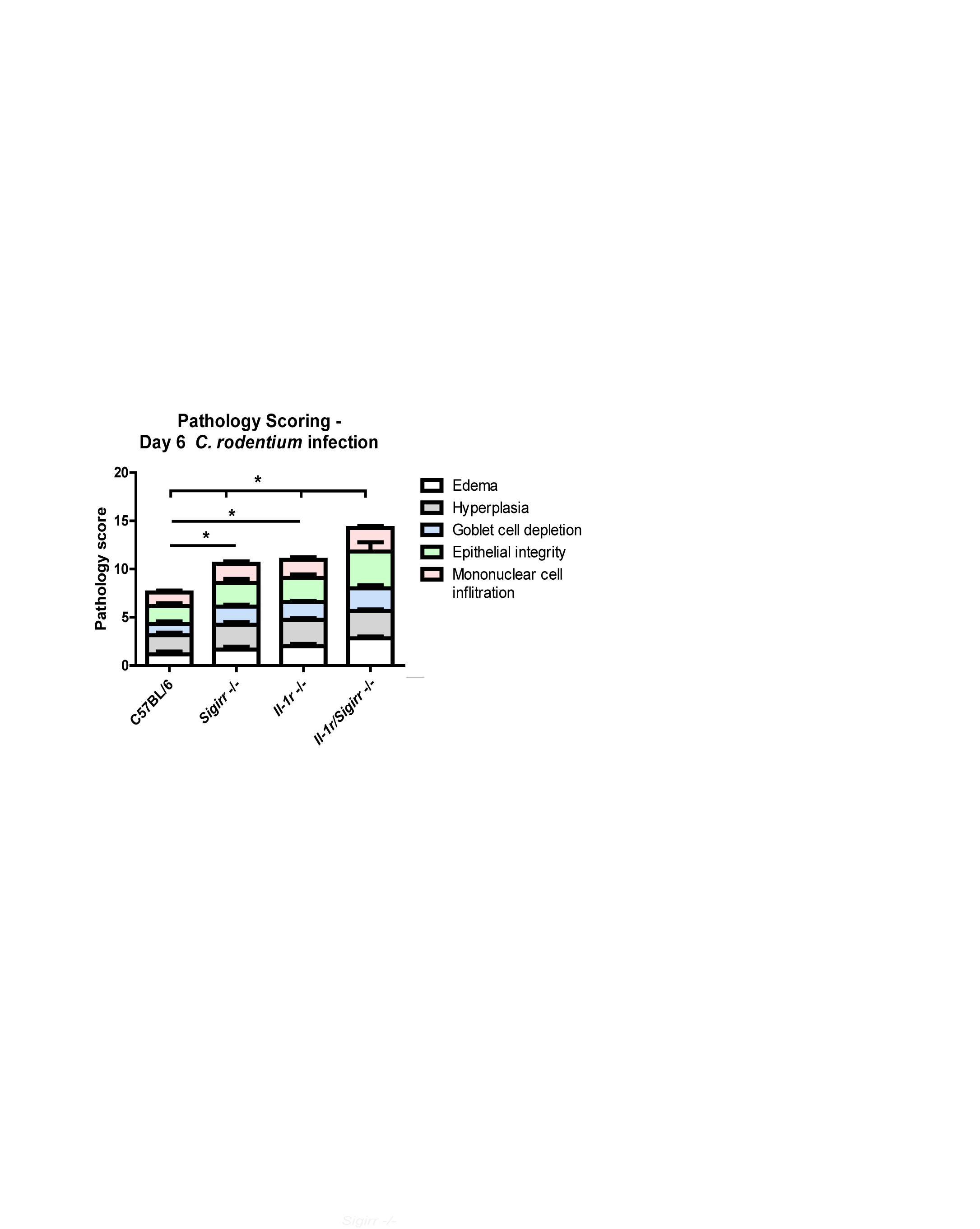

Supplement: Figure S5 — Total pathology score for Il-1r −/− and Il-1r/Sigirr −/− mice infected with C. rodentium for 6 days. Il-1r/Sigirr −/− mice exhibit increased pathological damage compared to Sigirr −/− and Il1r −/− mice. All three groups of mice suffered from greater damage compared to C57Bl/6 mice. Results are pooled from 2–4 independent infections with n = 3–4 per group. Error bars = SEM (Student t test, *P<0.05,) (TIF) [file ppat.1003539.s005.tif]
